# An Expanded Polyproline Domain Maintains Mutant Huntingtin Soluble *in vivo* and During Aging

**DOI:** 10.3389/fnmol.2021.721749

**Published:** 2021-10-15

**Authors:** Maria Lucia Pigazzini, Mandy Lawrenz, Anca Margineanu, Gabriele S. Kaminski Schierle, Janine Kirstein

**Affiliations:** ^1^Department of Molecular Physiology and Cell Biology, Leibniz Research Institute for Molecular Pharmacology in the Forschungsverbund Berlin e.V. (FMP), Berlin, Germany; ^2^NeuroCure Cluster of Excellence, Charité Universitätsmedizin Berlin, Berlin, Germany; ^3^Advanced Light Microscopy, Max-Delbrück Centrum for Molecular Medicine (MDC), Berlin, Germany; ^4^Molecular Neuroscience Group, Department of Chemical Engineering and Biotechnology, University of Cambridge, Cambridge, United Kingdom; ^5^Department of Cell Biology, University of Bremen, Bremen, Germany

**Keywords:** *C. elegans*, huntingtin (HTT), polyQ, proline, aggregation, fluorescence life time imaging, aging, behavior - genetics - molecular

## Abstract

Huntington’s disease is a dominantly inherited neurodegenerative disorder caused by the expansion of a CAG repeat, encoding for the amino acid glutamine (Q), present in the first exon of the protein huntingtin. Over the threshold of Q39 HTT exon 1 (HTTEx1) tends to misfold and aggregate into large intracellular structures, but whether these end-stage aggregates or their on-pathway intermediates are responsible for cytotoxicity is still debated. HTTEx1 can be separated into three domains: an N-terminal 17 amino acid region, the polyglutamine (polyQ) expansion and a C-terminal proline rich domain (PRD). Alongside the expanded polyQ, these flanking domains influence the aggregation propensity of HTTEx1: with the N17 initiating and promoting aggregation, and the PRD modulating it. In this study we focus on the first 11 amino acids of the PRD, a stretch of pure prolines, which are an evolutionary recent addition to the expanding polyQ region. We hypothesize that this proline region is expanding alongside the polyQ to counteract its ability to misfold and cause toxicity, and that expanding this proline region would be overall beneficial. We generated HTTEx1 mutants lacking both flanking domains singularly, missing the first 11 prolines of the PRD, or with this stretch of prolines expanded. We then followed their aggregation landscape *in vitro* with a battery of biochemical assays, and *in vivo* in novel models of *C. elegans* expressing the HTTEx1 mutants pan-neuronally. Employing fluorescence lifetime imaging we could observe the aggregation propensity of all HTTEx1 mutants during aging and correlate this with toxicity via various phenotypic assays. We found that the presence of an expanded proline stretch is beneficial in maintaining HTTEx1 soluble over time, regardless of polyQ length. However, the expanded prolines were only advantageous in promoting the survival and fitness of an organism carrying a pathogenic stretch of Q48 but were extremely deleterious to the nematode expressing a physiological stretch of Q23. Our results reveal the unique importance of the prolines which have and still are evolving alongside expanding glutamines to promote the function of HTTEx1 and avoid pathology.

## Introduction

Huntington’s disease (HD) is a monogenic hereditary neurodegenerative disorder caused by the expansion of a glutamine (Q, CAG) stretch in the first exon of the protein huntingtin (HTT) (The Huntington’s Disease Collaborative Research Group, 1993). HD is part of a class of nine disorders known as polyglutamine (polyQ) diseases that all share the same pathological culprit in a polyQ expansion. In each polyQ disorder, this unusually long Q stretch is, however, located in different genes, expressing proteins of different functions and generating different pathologies. In all nine disorders, CAG repeats become pathological only above a defined threshold, which is specific for each disease, albeit similar (Zoghbi and Orr, 2000). For HD, the disorder is fully penetrant above the threshold of Q ≥ 39; for intermediate alleles of 35 < Q < 39 the disease might manifest late in life, whilst for Q ≤ 35, there is no pathology. There is also an inverse correlation between the age of onset and severity of the disorder, with larger CAG expansions promoting earlier onset and graver symptoms (Wright et al., 2020). The CAG repeat, and its following CCG triplet expansion, encoding for a polyproline (polyP) stretch, are also highly unstable sequences (Lee et al., 2010). CAG and CCG present with somatic variability within and throughout brain regions, potentially contributing to symptom and onset irregularities (Vuillaume et al., 1998; Swami et al., 2009). HD manifests itself initially with psychiatric and cognitive symptoms, developing into motor impairments, which lead to death within 15–20 years of onset. From a cytopathological perspective, HD causes atrophy of the basal ganglia, a result of the extensive loss specifically of medium spiny neurons of the striatum (Walker, 2007).

Huntingtin is a large ubiquitous protein of 3144 amino acids without a fully defined function. HTT is involved in transcription, translation, synaptic signaling, vesicle transport, mitochondrial energy production, macro-autophagy and many other processes; its large interaction partner network also suggests a general scaffolding role (Saudou and Humbert, 2016). Alternative splicing and several caspase-mediated cleavage events reduce the full length HTT into progressively smaller fragments, key of which is the ≈100 amino acid N-terminal first exon of HTT (HTTEx1), where the polyQ expansion is located (Graham et al., 2006; Neueder et al., 2018). HTTEx1 is responsible for the formation of the pathology’s hallmark amyloids structure, or inclusion bodies, found in both the cytoplasm and nucleus of HD patient’s neurons (Mangiarini et al., 1996; Davies et al., 1997; DiFiglia et al., 1997). HTTEx1 has been repeatedly shown to aggregate and form typical amyloid structures *in vitro* (Scherzinger et al., 1997). Whether these insoluble aggregated structures are at the base of neuronal toxicity is however still controversial. Aggregation load does not consistently correlate with cytotoxicity and aggregates themselves may have a protective role by sequestering mutant soluble HTT (Saudou et al., 1998; Arrasate et al., 2004). Soluble expanded mutant HTT might exhort neurotoxic processes through both a gain of toxic function and/or a loss of physiological function. Indeed intermediate or oligomeric species, on pathway to fibril formation, have also been implicated in toxicity, yet their neurotoxic mechanism is poorly understood (Poirier et al., 2002; Sánchez et al., 2003).

**FIGURE 1:**
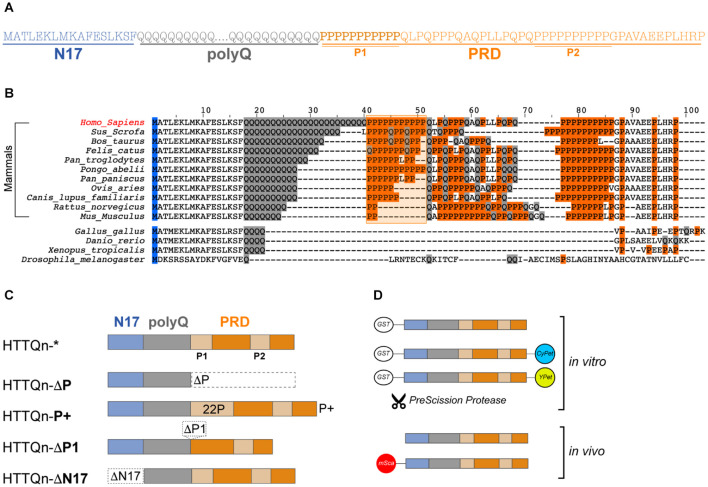
Huntingtin exon 1: Sequence, evolution, key domains and their variations. **(A)** Amino acid sequence of human HTTEx1 and its domains: the first 17 amino acids (N17 – light blue), the variable polyglutamine region (polyQ – gray) and the proline rich domain (PRD – orange), which includes two stretches of pure polyproline (P1 and P2). **(B)** Alignment of human huntingtin HTTEx1 and of selected species. Amino acids relevant for this study are highlighted: glutamines (Q) in gray, prolines (P) and the P1 region, shaded and contoured, in orange. **(C)** Schematics of the HTTEx1 mutants generated for this study: HTTQn* is the non-variated HTTEx1, in HTTQn-**ΔP** the proline rich domain (PRD) was deleted; in HTTQn-**P+** the first proline stretch (P1) of the PRD was expanded from 11 to 22 prolines; in HTTQn-**ΔP1** the whole first proline stretch (P1) has been deleted; and in HTTQn-**ΔN17** the first 17 amino acids have been deleted. Domains are color coded throughout the study: N17 in light blue, polyQ in gray and PRD in orange. Regarding the polyQ, all mutants contain either a non-expanded and physiological Q23 or pathogenic Q48 stretch. **(D)** Graphical representation of the fusion constructs used in this work: for the *in vitro* investigations, HTT constructs are tagged N-terminally to a removable GST-tag, and C-terminally to either CyPet or YPet fluorophore, or nothing. For the *in vivo* analysis, HTTEx1 is either fully untagged, or fused N-terminally with a nematode adapted mScarlet fluorophore.

Amyloid formation is promoted by the polyQ expansion, which folds into an antiparallel β-sheet structure held together by the hydrogen bonding of interdigitating glutamine side chains, in the form of a polar zipper (Perutz, 1995). Within HTTEx1, the core polyQ domain is found encased between two domains: an N-terminal stretch of 17 amino acids (N17) and a C-terminal proline-rich domain (PRD), of approximately 50 amino acids (Figure 1A). The highly conserved N17 adopts an amphipathic α-helix structure capable of interacting with lipid membranes, chaperones and other HTT molecules; its post-translational modifications (PTMs) modulate HTT’s function and translocation, and enhance its aggregation potential, with repercussions on toxicity (Arndt et al., 2015; Baias et al., 2017). The evolutionary newer PRD adopts a left-handed type II polyproline helix (PPII) (Darnell et al., 2009) and was reported to delay fibril formation and assert a protective function (Bhattacharyya et al., 2006; Dehay and Bertolotti, 2006). The PRD is composed of two stretches of pure proline repeats (P1 and P2) separated by a proline/glutamine rich region (Figure 1A). Proline is a unique amino acid due to the cyclical nature of its side chain: polyP stretches impose limited conformational flexibility and thus structural rigidity (Williamson, 1994). Yet, proline stretches also present both an accessible hydrophobic surface and good hydrogen bonding sites allowing for rapid and modulated protein interactions, with physiological relevance in transcription, cell motility and signal transduction processes (Kay et al., 2000; Moradi et al., 2011). Single prolines are found copiously dispersed in intrinsically disordered regions to impose both flexibility and function, but natural selection has also placed polyP at the edges of aggregation-prone sequences to protect them from self-association, ultimately acting as gate keepers (Reumers et al., 2009; Lee et al., 2014). The PRD and the N17 flanking domains of HTTEx1 propagate their own conformational and physicochemical features into the polyQ stretch, influencing its structural versatility and aggregation propensities (Caron et al., 2013; Urbanek et al., 2020b). But while the role of the N17 is well established, much less is understood about the function of the PRD, and especially of its two repeated mono-proline stretches.

In this study, we aimed to show the key and contrasting roles of the flanking domains in modulating aggregation and toxicity *in vitro* and *in vivo*, and specifically the involvement of the newly evolved proline repeat immediately adjacent the polyQ (P1). We hypothesized that in mammals the P1 domain has been evolving and elongating in parallel to the expansion of the polyQ stretch, with a clear purpose. The lengthening of the P1 would be able to counteract the aggregation propensity of the expanded polyQ to ultimately maintain HTT’s functionality in the evolution of the nervous systems. We thus decided to empirically expand the P1 and investigate its effect on aggregation and toxicity of both pathogenic or physiologic HTTEx1. By generating mutants lacking either flanking region, we first confirmed previous *in vitro* findings of the effects of the N17 and the PRD domains. When doubling the size of the P1 stretch, we uncovered *in vitro* an increase in aggregation propensity, promoted by the PRD. This heightened aggregation propensity was, however, not replicated *in vivo* and instead HTTEx1 mutants with an expanded polyP remained soluble over time. To understand the contribution of the flanking domains in a physiological and cellular context, we generated the first *Caenorhabditis elegans (C. elegans)* strains expressing pan-neuronally the HTTEx1, complete or with its mutated flanking domains. Employing fluorescence lifetime imaging (FLIM) we were able to distinguish the formation of soluble *vs* oligomeric *vs* aggregated species *in vivo* and during aging, and correlate these to toxicity at an organismal level. Our results confirmed the deleterious effects imposed by a missing PRD, while highlighting the benefits of possessing an expanded polyP domain in the presence of an expanded polyQ. To our surprise, a long polyP stretch flanking a physiological polyQ sequence was instead highly deleterious to the nematode’s fitness. We conclude that in their evolutionary expansion, HTTEx1’s glutamine and proline tracts are poised to find a functional balance, while avoiding the risk of pathology.

## Materials and Methods

### Gene Alignment and Species Comparison

FASTA sequences were obtained by searching the NCBI Protein Database after performing a BLAST search against the human HTTEx1 sequence. Alignments and coloring of selected species sequences were generated manually in Jalview (v. 2.11.1.0) (Waterhouse et al., 2009). Sequence accession codes are as follow: *Homo sapiens* (NP_002102.4), *Sus scrofa* (NP_999129.1), *Bos taurus* (XP_002688476.2), *Felis catus* (XP_023109234.1), *Pan troglodytes* (XP_016806693.2), *Pongo abelii* (XP_024101641.1), *Pan pansiscus* (XP_003813027.3), *Ovis aries* (NP_001136110.1), *Canis lupus familiaris* (XP_038517621.1), *Rattus norvegicus* (NP_077333.2), *Mus musculus* (NP_034544.1), *Gallus gallus* (XP_420822.3), *Danio rerio* (NP_571093.1), *Xenopus tropicalis* (XP_031751173.1), and *Drosophila melanogaster* (NP_651629.1).

### Generation, Expression and Purification of *in vitro* Non-tagged HTTEx1 Mutant Constructs

HTTEx1 constructs containing the flanking region variations were synthetized in pUC57 plasmids. ΔP, P+ and ΔN17 constructs were synthetized by BioCat GmbH (Heidelberg), while non-variated HTTEx1 and ΔP1 mutant were synthetized by GenScript United States Inc. (Piscataway, NJ, United States). In all plasmids, the HTTEx1 sequence was preceded by the restriction enzyme sites *SacII, PstI* and *BamHI* and succeeded by the sites *XhoI, NotI* and *MluI*, in this order. All sequences ended with a stop codon and were synthetized with a stretch of either Q23 or Q48. HTTEx1 mutants were cloned into a pGEX-6P-1 backbone between *BamHI* and *XhoI*, and transformed into BL21-CodonPlus (DE3)-RIPL competent cells (Agilent, #230280). Expression of a fully-grown culture was induced by the addition of 1 mM IPTG and left at 20°C overnight, shaking at 200 rpm. Cells were harvested and resuspended in lysis buffer (50 mM NaH_2_PO_4_, 5 mM Tris, 150 mM NaCl, 1 mM EDTA, pH 8.0) supplemented with 2 mM phenylmethylsulphonyl fluoride, DNase and cOmplete^TM^ Protease Inhibitor Cocktail tablets (Roche). Cells were lysed by shredding using the LM10 microfluidizer set at 18.000 psi, for 5 consecutive cycles (Microfluidics International Corporation). After lysis, TritonX-100 was left to dissolve to a final concentration of 1% (v/v) and the lysate was centrifuged at 30.000 × *g* for 40 min at 4°C (Sorvall LYNX 4000, Thermo Scientific). The resulting supernatant was mixed with pre-washed glutathione sepharose 4B beads (GE Healthcare) and incubated at 4°C for 90 min rolling. The lysate was then applied to a polypropylene column (Qiagen, #34964) and washed twice with wash buffer (50 mM NaH_2_PO_4_, 5 mM Tris, 150 mM NaCl, 1 mM EDTA, 0.1% (v/v) Triton-X-100, pH 8.0). After washing, beads were incubated for 30 min at RT with elution buffer (50 mM NaH_2_PO_4_, 5 mM Tris, 150 mM NaCl, 1 mM EDTA, 20 mM reduced glutathione, pH 8.6). The resulting eluate was dialyzed against storage buffer (50 mM Tris, 150 mM NaCl, 1 mM EDTA, 5% (v/v) glycerol, pH 7.4) for 16 hours at 4°C. Proteins were assessed for purity via SDS-PAGE analysis and stored at −80°C.

### Cloning and Purification of CyPet/YPet-Tagged HTTEx1 Mutant Constructs

HTTEx1-CyPet/YPet mutants were created by modifying the plasmid sequence of the GST-HTTEx1Q_48/23_ -CyPet and GST- HTTEx1Q_48/23_ -YPet constructs (Scior et al., 2018). To remove the stop codon and add appropriate restriction sites, plasmids of HTTEx1-P+ and ΔP1 mutants were amplified by PCR using the forward primer: *cgccgcggatccatggcgaccctggaaaagctg*; and reverse primer: *ccgccgctcgagtggtcggtgcagcggctcctc*. ΔP and ΔN17 mutants without an AUG codon at the C-terminus of HTTEx1 sequence were synthetized by BioCat GmbH (Heidelberg). PCR or plasmid-derived mutant DNA were sub-cloned into the GST-CyPet or GST-YPet backbone, between *BamHI* and *XhoI*. Expression and purification of HTTEx1-CyPet/YPet constructs were performed as described above for non-tagged constructs.

### *In vitro* Aggregation Assays

Prior to all *in vitro* experiments, GST-HTTEx1-X proteins were thawed on ice, ultra-centrifuged at 154.000xg for 45 min at 4°C (Rotor TLA55, Beckman Coulter) and their concentration determined via Bradford assay. Unless otherwise stated, aggregation was performed at 30°C and 450 rpm shaking in aggregation buffer (30 mM Tris–HCl, 150 mM NaCl, 1 mM EDTA, 1 mM DTT, pH 7.4). Proteins were subjected to Tris-Glycine 10% SDS–PAGE and stained with InstantBlue protein stain (Sigma) or employed for Western blotting.

### Atomic Force Microscopy Sample Preparation and Imaging

3 μM HTTEx1 and 14 U PreScission protease (PP) (GE Healthcare) were incubated in a volume of 20 μl for either 2, 4 or 24 h. The whole sample volume for each time point was spotted onto a freshly cleaved mica surface and left to dry at RT for 10 min. Samples were washed with 40 μl ddH_2_O four times and left to dry covered for 48 hours at RT. Imaging was performed using the digital multimode NanoWizard II (JPK) atomic force scanning microscope operating in intermittent-contact air tapping mode and image analysis was performed with JPK Software analysis package (Bruker Nano GmbH).

### Semi-Denaturing Detergent Agarose Gel Electrophoresis

Semi-Denaturing Detergent Agarose Gel Electrophoresis (SDD-AGE) gels for detection of amyloids were performed based on previously described protocols (Halfmann and Lindquist, 2008), with an HTTEx1 starting concentration of 3 μM. At 4 and 24 h after addition of PP, 4x Laemmli buffer (LB) without SDS (0.125 M Tris–HCl, 20% glycerol, 10% 2-mercaptoethanol, 0.004% bromophenol blue) was added to the aggregated reaction and incubated at RT for 7 min. Samples were immediately loaded on a 1.8% agarose gel in 1xTAE containing 0.1% SDS and subjected to 22V for 20 h on ice in a cold room in 1xTAE buffer supplemented with 0.1% SDS. TEA agarose gels were subsequently laid onto a same-sized nitrocellulose membrane with 0.2 μm pore size (GE Healthcare, #10600004) and placed onto a 25-piece stack of thick Whatman blotting paper (GB002). Paper, gel and nitrocellulose stack were covered with a wet wick reaching into two PBS reservoirs, and a heavy weight was applied on top of the ensemble. Proteins were left to transfer via capillary action for 24 h at RT. The membrane was then blocked in 5% skim milk in TBS-T and incubated overnight at 4°C with primary antibodies in 3% skim milk in TBS-T. Membranes were incubated with the primary anti-HTTEx1 antibodies EPR5526 (1:2000, Abcam) and MW8 (1:2000; DSHB) and subsequently probed with secondary antibodies conjugated to horseradish peroxidase (HRP) against rabbit or mouse (1:10,000; #31460 and #31444 respectively, Thermo Fisher). Signals were developed with Pierce ECL Western Blotting Substrate (#32209, Thermo Fisher) and imaged on a Lumi-Imager F1 system employing LumiAnalyst software (Boehringer Mannheim GmbH).

### Filter Retardation Assay

Filter retardation assay (FRA) was performed as previously described (Ast et al., 2018b), utilizing a final protein concentration of 1.5 μM. The addition of PP started the aggregation process. At the indicated time points at 0, 4, 8 and 24 h after addition of PP, an aliquot was taken from each sample and flash frozen in liquid nitrogen. Before blotting, samples were mixed 1:1 v/v with a stop solution (4% SDS, 100 mM DTT). In a dot blot apparatus attached to a pump, whole samples were loaded on and filtered simultaneously through a cellulose acetate membrane with 0.2 μm pores (OE66, GE Healthcare, #10404180) pre-soaked in TBS buffer containing 1% SDS. All blot wells were washed twice with TBS plus 0.1% SDS and membranes were then blocked in 5% skim milk in TBS-T. Fibrils retained on the membrane were detected by probing with both anti-HTTEx1 primary antibodies EPR5526 (1:1000; Abcam) and MW8 (1:2000; DSHB). Anti-rabbit HRP secondary antibody (1:10,000; #31460, Thermo Fisher) was used to probe HTTEx1 EPR5526 and IRDye680 anti-mouse (1:10.000; #926-68070 LI-COR Biosciences) was used for detection of MW8. HRP signals were developed with Pierce ECL Western Blotting Substrate (#32209, Thermo Fisher) and imaged using the OdysseyFC imaging system (LI-COR Biosciences).

### Sodium Dodecyl Sulfate Stability Assay

1 μM HTTEx1 was left to aggregate for 24 h after addition of PP. At that point, either 0,1% SDS or 2% SDS (v/v) was added to each sample and tubes were heated at 60°C for 10 min. Samples were then spun at 154.000 × g for 20 min at 4°C (Rotor TLA55, Beckman Coulter). Subsequently, 80% of supernatant was carefully transferred to low-binding tubes containing 4xLB without SDS, gently mixed, and finally boiled for 5 min at 100°C. Samples were subjected to 10% SDS–PAGE gels in Tris-Glycine buffer and transferred on a nitrocellulose membrane via semi-dry blotting (Trans-Blot Turbo System, BioRad). Western blotting procedures and development were performed as described above and membranes were probed with the primary HTTEx1 antibody 5TF1-1C2 (1:5000, Merck) and HRP-conjugated secondary anti-mouse antibody (1:10,000, #31444, Thermo Fisher). Quantification of the intensity measurements of the blots were performed using the software Fiji/ImageJ (Schindelin et al., 2012).

### Förster Resonance Energy Transfer Assay

Förster resonance energy transfer (FRET) assay was performed as previously described (Ast et al., 2018a; Scior et al., 2018). Purified CyPet-YPet pairs of each HTTEx1 mutant were diluted in FRET aggregation buffer (30 mM HEPES-KOH, 150 mM KCl, 5 mM MgCl_2_, 1 mM DTT, pH 7.4) in equimolar ratios to yield a final concentration of 3 μM. For spiking experiments, equimolar ratio of tagged *vs* untagged mutants was used, still maintaining a final concentration of 3 μM. For experiments with Q48ΔP titrations, the ratio of untagged ΔP was gradually increased while the CyPet-YPet decreased, but always maintaining a final concentration of 3 μM. For experiments including HTTEx1Q23 mutants, concentrations were increased to a total of 40 μM to accelerate aggregation. 5 mM ATP, 3 mM phosphoenolpyruvate and pyruvate kinase were added to the reaction, and a final addition of 14 U of PP initiated the aggregation process. Reactions were split into triplicates in a black 384-well plate. Using a Tecan F200Pro plate reader (Tecan Trading AG), fluorescence signals were recorded every 30 min for constructs containing Q48, and every hour for constructs containing Q23. Experiments were performed for a minimum of 15 and a maximum of 660 h. Fluorescence of the donor CyPet constructs (DD) was measured at 430/485nm emission/excitation; the YPet acceptor (AA) was measured at 485/530nm emission/excitation; and the FRET channel (DA) was collected at 430/530nm emission/excitation. For data processing, the background fluorescence signal of each channel was removed from all respective values. The FRET signals were corrected for donor bleed-through (cD) and acceptor cross excitation (cA) by using donor- or acceptor-only values. This was performed for each mutant independently. A final sensitized FRET emission was normalized to the acceptor. The apparent FRET efficiency (EApp), expressed in percentage values, was therefore calculated as: E = (DA-cD^∗^DD-cA^∗^AA)/AA (Jiang and Sorkin, 2002). All EApp values were expressed in percentage, set to zero and normalized to HTTEx1Q48-CyPet/YPet signals.

### Cloning of Mutant HTTEx1 Constructs for Nematode Expression

Constructs for generation of the HTTEx1 *C. elegans* strains for fluorescence lifetime imaging were created by modifying the neuronal wrmScarlet::Aβ_1–42_ plasmid (Gallrein et al., 2021). Gibson assembly (E2611L, New England Biolabs) was used to remove the Aβ peptide and to create a backbone including new restriction enzyme sites for addition of HTTEx1. A linear backbone comprising the neuronal promoter, resistance gene, origin of replication and the *unc-54*-3′UTR regulatory regions was generated using the following forward and reverse primers: *ctgcagtgcattggataagaatgcggccgcTAGCTCGAGATACCCAGATCATATGAAACGG* and *acgcgtcgcgtcctccccgcgg CCCGGTCGTcgtcgtcgtcgatgccgtc.* The sites *NotI* and *PstI*, separated by the base pairs *tgcattggataagaat*, were introduced at the 5′-end of this amplicon, and the *SacII* and *MluI* sites, interspaced by the base pairs *ggaggacgc*, were added to the 3′-end of the plasmid. A smaller PCR product including the sequences for the *hsp3*^IRES^ region and the mScarlet fluorophore were amplified using the forward and reverse primers: *ggcatcgacgacgacgacgaccgggccgcggggaggacgcgacgcgtTAAACCGGT TGCTCTCCCTC* and *catatgatctgggtatctcgagctagcggc cgcattcttatccaatgcactgcagGCTAGCCTTGTAGAGCTCGTC*. The resulting plasmid was used as starting point to create all HTTEx1 nematode constructs. This plasmid was further employed, unchanged, to create a control strain that would express only the mScarlet fluorescent protein. Introduction of the two moieties of HTTEx1 was performed in two steps. First, HTTEx1 was introduced downstream of the IRES-mScarlet sequence between *NotI* and *PstI*. Then, a second HTTEx1 sequence, identical to the first, was introduced downstream of the *rgef-1* promoter and before the IRES-mScarlet sequence, between *SacII* and *MluI*. All cloning was confirmed via Sanger sequencing (LCG Genomics) to ensure constructs included two correct HTTEx1 moieties. For detailed genotype of each *C. elegans* strain generated please refer to Supplementary Table 1.

### Generation and Maintenance of HTTEx1 *C. elegans* Strains

All transgenic strains were generated by microinjection following previously established protocols (Mello et al., 1991). For each HTTEx1 construct, 5–20 ng/μl of plasmid DNA plus filler DNA to 200 ng/μl (DNA Ladder, #SM0311, Thermo Fisher) were injected into 20 wild type N2 nematodes, progeny was scored for red fluorescence and positive nematodes singled out. Extra-chromosomal arrays of HTTEx1 nematodes were integrated via UV irradiation, following the previously described procedure, and 100% transmitting positive lines were backcrossed four times against N2 wild type. For imaging experiment, non-integrated HTTEx1 nematodes lines were employed, while phenotypic assays were conducted with integrated and backcrossed lines. All *C. elegans* strains were maintained on nematode growth media (NGM) plates seeded with *E.coli* OP50 at 20°C, according to standard practice (Stiernagle, 2006).

### Confocal Imaging of Live *C. elegans* HTTEx1 Models

Nematodes were mounted on 3% agarose pads precast on microscopy slides and were anesthetized with 2 mM levamisole (#A4341, AppliChem GmbH). Imaging of the mScarlet (561/630 ex/em) was performed on an inverted Zeiss LSM710 ConfoCor3 and using Zeiss C-Apochromat 10x/0.3W or PLAN-Apochromat 63x/1.40 Oil DIC M27 objectives (Carl Zeiss AG). Image processing and analysis was performed with Fiji/ImageJ.

### Time Correlated Single Photon Counting Fluorescent Lifetime Imaging of HTTEx1 *C. elegans* Strains and Image Analysis

Fluorescence lifetime imaging of HTTEx1 *C. elegans* was performed as described in detail previously (Pigazzini et al., 2020). Briefly, nematodes were age synchronized via egg laying and grown on NGM plates seeded with OP50 at 20°C, for either 4 or 10 days. Nematodes were mounted on 3% agarose pads precast onto microscopy slides and were anesthetized with either 2 mM levamisole for day 4 nematodes, or 0.5 mM levamisole for day 10 nematodes. TCSPC-FLIM was performed on an inverted Zeiss AxioObserver.Z1 (Carl Zeiss AG) confocal microscope connected to a B&H DCS-120 SPC-150 TCSPC acquisition hardware and B&H SPC830-SPC Image software (Becker & Hickl GmbH). Images were acquired using a Zeiss ApoE 63x/1.20 Oil objective and with 1.5x magnification. mScarlet was excited with a 50 MHz 60 ps pulse width 561 nm laser. The emission was collected through a combination of a 575 long pass and a 628/40nm bandpass filter. Photons were collected for 120 s per nematode. Intensity decay fitting was performed with FLIMfit software (v 5.1.1) (Warren et al., 2013) using reference deconvolution (measured with an erythrosine B solution in water). Although the decays showed a multiexponential profile, to simplify the data interpretation we performed an image-wise fit using a single exponential model that accounts for the average lifetime.

### Lifespan Assay

*C. elegans* were age synchronized by egg laying for 4 hours at 20°C and kept at 20°C throughout the whole experiment. On day 3, 120-180 L4 nematodes were divided onto 5 NGM plates seeded with OP50. *C. elegans* were counted daily and scored as alive, dead, or censored. Nematodes were passaged daily to fresh plates until day 10, after which they were only passaged if necessary. Three biological repeats for each strain were performed. The Kaplan Mayer method was used to calculate and compare the survival distribution of two or more groups. A log-rank (Mantel-Cox) test was used to estimate significance. Lifespan results were calculated using the online OASIS 2 tool (Han et al., 2016); results from this analysis are available in Supplementary Table 3.

### Progeny Assay

Nematodes were age synchronized by egg laying for 4 h at 20°C and kept at 20°C throughout the whole experiment. On day 2 after hatching, 20 nematodes were placed individually on an NGM plate seeded with OP50. Once egg laying began, nematodes were passaged daily to a fresh plate, and eggs were left to hatch at RT for 24 hours before being counted. Progeny was counted for five days. Three independent biological repeats for each strain were performed and the average ± SD of all triplicates combined was used for statistical analysis.

### Chemotaxis Assay

*C. elegans* were age matched via alkaline hypochlorite solution treatment (Porta-de-la-Riva et al., 2012). Chemotaxis assay was performed as described previously (Margie et al., 2013). Briefly, on day 4 after synchronization, nematodes were collected from synchronized NGM plates and washed 5x times with M9 solution to fully remove OP50. Nematodes were left to starve in M9 at 20°C for 30 minutes before commencing the assay. Nematode pellet was then separated onto plates pre-prepared with either an attractant (1% benzaldehyde, v/v), a repellent (50 % benzaldehyde, v/v) or a control (50% EtOH, v/v) solution. All test solutions were dissolved 1:1 in 500 nM sodium azide. 2 μl of chemotactic substance or control were placed on opposing quadrants of a single plate divided into four regions. 50-150 nematodes were pipetted at the center of a plate and left to crawl towards or away from the chemotactic test substance. The assay was performed at 20°C for 2 h in the dark, after which plates were placed at 4°C for a further 2 h to stop nematodes from moving. Nematodes in each quadrant were counted and a chemotaxis index (CI) was calculated as # of nematode in test quadrants minus # of nematodes in control quadrants, divided by total number of nematodes that left the center. Three independent biological repeats for each strain were performed and the average ± SD of each CI was then calculated and used for statistical analysis.

### Thrashing Assay

*Caenorhabditis elegans* were age synchronized by egg laying for 4 h at 20°C and kept at 20°C throughout the whole experiment. On either day 4 or 10, a single nematode was picked into 200 μl of M9 liquid and left to acclimatize for 30 s, before manually counting the number of thrashes performed in the subsequent 30 s. A single thrash is defined as a complete movement from minimum to maximum amplitude, and back. Cohorts of at least 10 nematodes each were analyzed in three independent biological replicates, and significance was calculated from these measurements.

### Statistical Analysis of FLIM Measurements and Phenotypic Assays

All statistical analysis was performed using Prism8 (GraphPad Software). Unless otherwise stated, to determine statistical significance for lifetime changes and most phenotypical assays, a one-way ANOVA followed by Tukey’s *post hoc* analysis method was employed.

## Results

### Proline Residues Are Expanding Alongside Glutamines

Huntingtin is well conserved throughout species (Gissi et al., 2006; Tartari et al., 2008). Prior investigations have revealed that glutamine residues are positionally conserved in deuterostomes and their numbers are increasing in size during evolution. First appearance of the *HTT* gene dates back millennia and orthology studies have identified a primordial version of *HTT* in amoeba and the first appearance of a single glutamine amino acid in the sea urchin. Significant lengthening of the glutamine stretch appears in vertebrates and correlates with increased sophistication of the nervous system (Tartari et al., 2008). Indeed, *HTT* has a clear function in brain development and is key in the early stages of gastrulation (Barnat et al., 2020). Removal of HTTEx1 is embryonically lethal, and conversely, longer glutamine stretches were reported to correlate with increase in gray matter volume (Nasir et al., 1995; Beste et al., 2008; Zuccato and Cattaneo, 2016; Conforti et al., 2018). While the importance of the polyQ sequence in development, growth and neuronal processing, as well as in aggregation and toxicity, is widely studied and accepted, the impact of the flanking regions has not been as extensively investigated. Similarly to prior investigations, we aligned the amino acid sequences of HTTEx1 homologues of selected species of vertebrates, according to their expanding polyQ domain (Figure 1B). The N17 is extremely conserved throughout these species, as is the final part of the PRD domain (PGPAVAEEPLHRP), comprising the second unusually long polyP stretch (P2). Importantly, the polyP domain immediately adjacent to the polyQ (P1), a sudden addition exclusively in mammals, is also highly conserved (Brandi and Polticelli, 2021). Furthermore, from this alignment it is noticeable that the P1 domain is expanding, similarly, and alongside the polyQ domain. Primates such as the orangutan (*Pongo abelii*), evolutionary closer to humans, present an uninterrupted stretch of prolines, while other mammals present a proline stretch that is interrupted by the presence of other amino acids such as glutamines or leucines (Barnes et al., 1994; Schmitt et al., 1995; Tartari et al., 2008). The importance and function of the elongated proline stretch, either interrupted or not, is however not fully characterized.

Our investigation aimed to understand the importance of the expanding P1 region, and in general of the PRD. We generated HTTEx1 mutants containing either physiological Q23 or pathological Q48, and variating flanking domains: we removed the whole PRD domain (ΔP), we arbitrarily doubled the polyproline domain from a typical 11P to a 22P (P+) stretch, we deleted the first polyP stretch only (ΔP1) and finally, we eliminated the first 17 amino acids (ΔN17) (Figure 1C). To investigate the impact of these HTTEx1 mutants *in vitro* we employed purified recombinant proteins, generated without or tagged with fluorescent probes. Similarly, to study the same mutants *in vivo* with regards to their aggregation propensity and toxicity, we leveraged the advantages of fluorescent fusion constructs expressed in the *C. elegans* model system (Figure 1D).

### Huntingtin Exon 1 Mutants Lacking a PRD Domain or Harboring an Expanded Proline Stretch Exhibit High Aggregation Propensities

The aggregation kinetics of HTTEx1 have been extensively investigated (Hoffner and Djian, 2015), but the contribution of the P1 region to the aggregation process is poorly characterized. We thus first set out to dissect the impact on aggregation of the P1, and generally the PRD, and hypothesized that an expanded polyP (P+) mutant could delay aggregation kinetics *in vitro*, while removing the P1 (ΔP1) would favor it. HTTEx1 aggregation speed correlates with increased polyQ length and fragment concentration (Yushchenko et al., 2018), following a nucleation/polymerization growth mechanism (Jayaraman et al., 2012). The N17 is responsible for initiation and accentuation of HTTEx1 oligomerization, through self-anchoring and PTMs (Gu et al., 2009; Thakur et al., 2009; Sivanandam et al., 2011). Conversely, the PRD has been shown to diminish the aggregation propensity *in vitro, ex vivo* and in yeast, slowing the formation of aggregates (Dehay and Bertolotti, 2006; Darnell et al., 2009; Shen et al., 2016). Our initial results also confirmed these profound impacts of the N17 and PRD flanking domains on the morphology and aggregation kinetics of HTTEx1: the contribution of the P1 itself however was more complex to discern.

**FIGURE 2:**
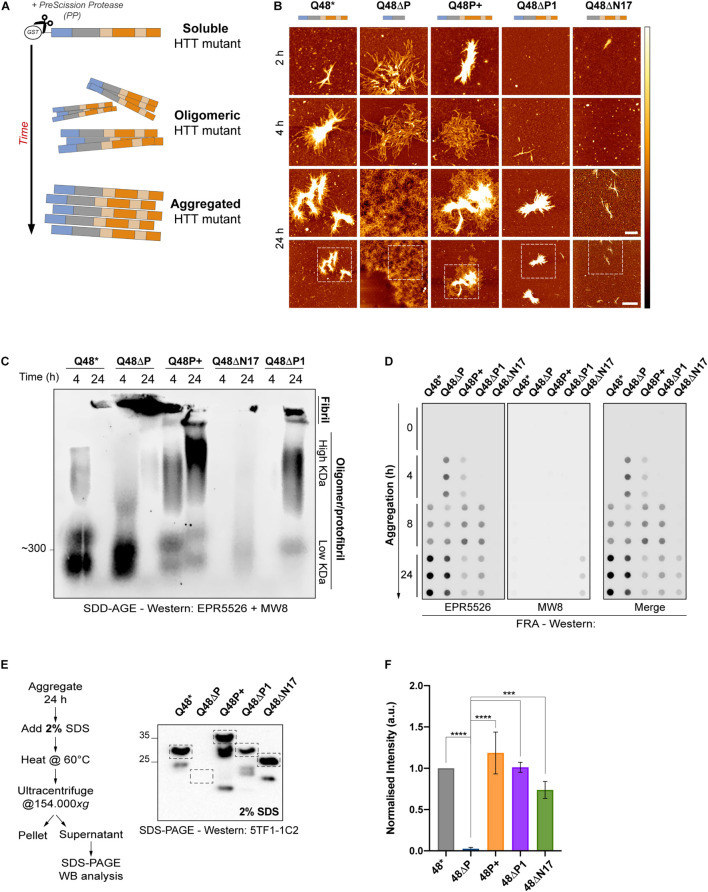
*In vitro* characterization of HTTEx1 mutant reveals a high aggregation potential for constructs without the PRD and with expanded prolines. **(A)** Schematic of the *in vitro* experimental set-up employed for all assays pictured herein. HTTEx1 mutants are purified fused to a GST-tag. Upon addition of the PreScission protease (PP), the GST-tag is cleaved and HTTEx1 moieties begin their aggregation process, transitioning from a soluble to an oligomeric and into a higher order aggregated structure. **(B)** AFM images of spontaneous HTTEx1 mutant aggregates and their unique morphology, at 2, 4 or 24 h after addition of PP. White to black color gradient represents 0 to 15 nm height. Scale bar for top three rows is 500 nm. Fourth row represents the wider field of view of scans in row three obtained at 24 h, scale bar is 2 μm. Dashed boxes represent the region of interest selected and magnified in the third row. **(C)** SDD-AGE of HTTEx1 mutants at 4 and 24 h after addition of PP. Lanes represent the different species of high molecular weight structures generated by each mutant: fibrillar species are at the top, trapped in the gel pockets, smears represent high molecular weight oligomeric/protofibrillar species, and lower signals are low molecular weight oligomers. Immunoblot stained with both the N17 specific EPR5526 and proline-binding MW8 antibodies. Gel shown here is representative of at least three independent replicates. **(D)** FRA showing the different time-dependent aggregation kinetics of HTT mutants. Samples were taken at 0, 4, 8 and 24 h after addition of PP and loaded in triplicates. Immunoblot was performed simultaneously with both the N17 specific EPR5526 antibody (left panel) or the MW8 proline region specific antibody (middle panel); right panel shows the merge of both. Blots are representative of at least three independent repeats. **(E)** Descriptive graphical abstract and Western blot analysis of the SDS stability assay. Blots represent the amount of soluble HTTEx1 present upon treatment with 2% SDS after 24 h of aggregation. Immunoblot stained for the polyQ-specific 5TF1-1C2 antibody. Bands utilized for quantification are boxed; lower bands represent truncated species and were not quantified. Shown here is one of three independent repeats. **(F)** Quantification of three independent SDS-SA, as per example shown in **(E)**. Values are normalized to Q48* and show mean ± SD; statistical significance was calculated using an ordinary one-way ANOVA followed by Tukey *post hoc* test and assuming 95% confidence interval (^∗∗∗^ = p < 0.0002; ^****^ = p < 0.0001).

We expressed HTTEx1 mutants as recombinant proteins N-terminally attached to a glutathione S-transferase (GST) tag (Supplementary Figure 1A). Excision of the GST-tag by the action of the PreScission protease (PP) liberates the HTTEx1 fragment from its fusion constructs and permits the start of aggregation (Figure 2A). We first confirmed that flanking domains had different, and opposing, impacts on the morphology of HTTEx1 mutants all containing a Q48 pathologic stretch. We employed atomic force microscopy (AFM) to visualize and compare at nanometer scale the aggregates formed by HTTEx1 mutants over time (Figure 2B). Previous imaging studies had already illustrated the capability of HTTEx1 to form visible fibrillar structures (Scior et al., 2018). After allowing mutants to aggregate spontaneously for 2, 4 or 24 h, we immediately noticed that at 2 h of aggregation, the ΔP mutant created large and intricate patterns of long and dispersed fibrillar fragments. Similar shapes were found at 4 h of aggregation, while at 24 h the size of the aggregates had overcome the field of view, stretching for > 10 μm in size (Figure 2B – second column). These fibrillar structures represent the aggressive aggregation propensity of the ΔP mutant, which is devoid of the protective or aggregation-delaying PRD domain. Conversely, the ΔN17 mutant, which lacks the aggregation initiating N17 domain, promoted the formation of only few fibrils, even over 24 h (Figure 2B – fifth column). Somewhat unexpectedly, P+ constructs also showed a propensity to form aggregates already at 2 h of aggregation, with large ensembles of fibrils at 4 h, which increasingly condensed and expanded at 24 h (Figure 2B – third column). These observations contrast with our expectation of an inhibitory role for the polyP on HTTEx1 aggregation, and instead the P+ mutant favored and accelerated aggregation compared to both the non-variated HTTEx1 and the ΔP1 mutant. In fact, while non-variated Q48^∗^ already formed aggregates at 4 h and larger structures at 24 h (Figure 2B – first column), the ΔP1 mutant exhibited aggregated bundles only at 24 h, highlighting the inability of the P1 to fully suppress the aggregation propensity (Figure 2B – fourth column).

Because of the counterintuitive outcomes of the AFM images for the P+ and ΔP1 mutants, we wanted to corroborate the morphological results with additional biochemical assays. To gain better insight of the different species of oligomers/aggregates formed by each mutant over time, we performed a semi-denaturing detergent agarose gel (SDD-AGE) followed by Western blotting (Figure 2C). Already at 4 h, non-variated Q48^∗^, ΔP and P+ mutants presented oligomeric species, that migrated at a high molecular weight of approximately 300 kDa, representing very different structures from the single globular moiety detectable at approximately 55 kDa (Figure 2C, Supplementary Figure 1A). At 24 h after aggregation initiation an intense signal was present for the ΔP mutant, as aggregated material that cannot migrate through the matrix of the agarose is retained in the pockets of the gel. Similar trapped material appeared for non-variated Q48^∗^ and for the ΔP1 mutant, suggesting that these aggerates are of a large and insoluble nature, confirming the AFM experiments (Figure 2B). For the P+ mutant, oligomeric species appeared in the middle of the gel lanes at both 4 and, more intensely, 24 h, possibly illustrating the presence of a more heterogenous population of aggregates; while again for the ΔN17 a very faint signal appeared only at 24 h, confirming its reluctance to aggregate. To further investigate the kinetics of aggregation of the HTTEx1 mutants we performed a filter retardation assay (FRA) (Ast et al., 2018b; Figure 2D). Samples from each aggregated mutant were analyzed at 0, 4, 8, and 24 h after aggregation initiation. At these time points, soluble material that was not trapped on the membrane passed through its pores, while aggregated moieties were retained on its surface. The FRA demonstrated that, once again, the ΔP mutant exhibited the fastest aggregation kinetics, generating a strong signal already 4 h after aggregation initiation. The P+ mutant similarly exhibited aggregated material early, while the non-variated Q48^∗^ and the ΔP1 aggregates appeared at around 8 h. Signals from ΔN17 mutant were only detected after 24 h of aggregation. Overall, the results of the FRA were in agreement with our data obtained from the AFM and SDD-AGE assays (Figures 2B,C). Both FRA and SDD-AGE are a qualitative addition to the AFM results, which provided only a snapshot of single timed events in the aggregation pathway. Quantification was hindered by the unavailability of a single antibody to detect all mutants in both assays. As no single antibody tested (EPR5526, 3B5H10, 5TF1-1C2, EM48, MW4, MW8, MW7) was able to recognize all HTTEx1 mutants simultaneously, a combination of two antibodies was therefore employed here: such a strategy prevented proper quantification of the signals (Figures 2C,D, Supplementary Figure 1C).

Finally, to assess the insolubility of aggregates formed by these mutants, we designed an assay to investigate their stability and resistance to sodium dodecyl sulfate (SDS) (Figures 2E,F, Supplementary Figure 1D). Addition of SDS to protein reactions or cell lysates is widely acknowledged as a method to promote solubilization, and thus allows to distinguish and separate soluble from truly aggregated, SDS-insoluble, species (Juenemann et al., 2015). HTTEx1 mutants were left to aggregate for 24 h and then treated with 2% SDS. Soluble versus insoluble fractions were separated by ultracentrifugation and the abundance of the soluble fraction, a readout of the fragility of the aggregates, was quantified by Western blotting (Figure 2E). Despite a high variability between repeats, the analysis revealed a clear trend. The ΔP mutant formed highly SDS-insoluble aggregates and no soluble fraction was detected, highlighting that all monomeric moieties are involved in the formation of these ΔP aggregates. Conversely, and regardless of its concentration, SDS was able to resolubilize larger species of all other mutants, similarly to the non-variated Q48^∗^ control.

From these *in vitro* results it emerged that the complete removal of the PRD exacerbated the aggregation capacity of HTTEx1. ΔP mutants rapidly form large and SDS-insoluble aggregates, incorporating quickly all available soluble protein. Expansion of the P1 did not alleviate aggregation, as hypothesized, and instead P+ mutants exhibited an increased aggregation propensity. However, the oligomeric species generated by the expanded P+ mutant, as illustrated by SDD-AGE and AFM assays, might be the product of long proline stretches that self-associate specifically *in vitro* and in the absence of other interactors. This self-binding might be transitory and thus easier to resolubilize upon treatment with SDS. Also, unexpectedly, the ΔP1 mutant failed to enhance the aggregation propensities of HTTEx1, and rather behaved like the non-variated Q48^∗^ throughout most biochemical assays. The ΔN17 mutant instead performed exactly as expected, promoting little aggregation over long time periods and thus highlighting the importance of the N17 domain in initiating aggregation.

### Deletion or Expansion of PRD Domains Alters Aggregation Kinetics Also for Short Q Variants

**FIGURE 3:**
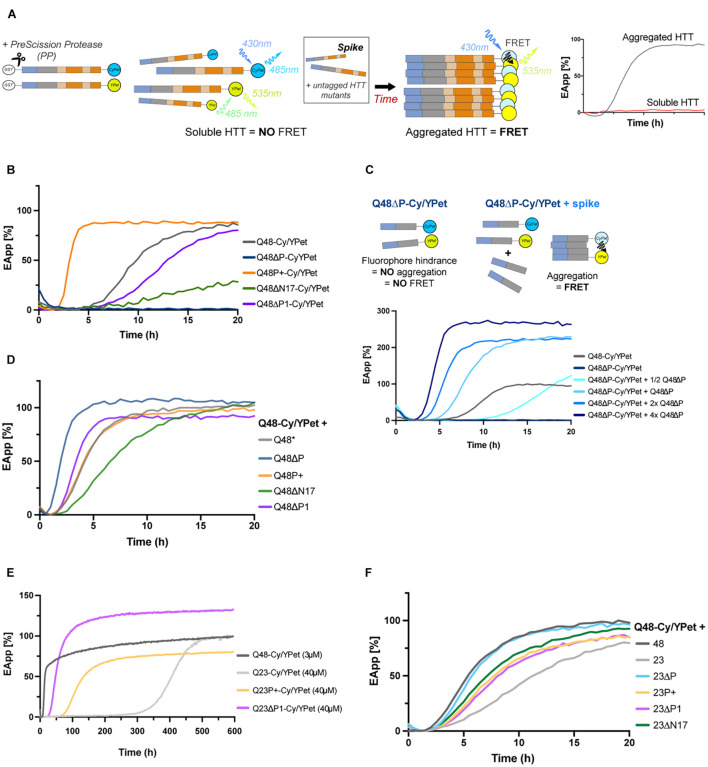
Deletion or expansion of PRD facilitates aggregation even for short polyQ variants. **(A)** Schematic of the FRET-based aggregation assay: upon addition of PP, CyPet/YPet tagged HTTEx1 mutants initiate aggregation, generating a readable and increasing FRET signal. In some experiments, the CyPet/YPet-tagged HTTEx1 moieties are mixed with non-tagged mutant HTTEx1, or ‘spiked.’ A fully aggregated reaction will be illustrated by a sigmoidal curve reaching a plateau over time, while soluble moieties will appear as flat lines. **(B)** FRET analysis of HTTEx1 mutants individually tagged with CyPet/YPet. The FRET signal of the non-variated Q48-CyPet/YPet serves as control (gray line). **(C)** FRET analysis of ΔP mutants: due to the steric hindrance imposed by the fluorophores (as illustrated in the cartoon schematic), readable FRET signals only appears when Q48ΔP-CyPet/YPet pair is spiked with untagged ΔP, with faster kinetics with increasing untagged Q48ΔP concentration. **(D)** FRET analysis of non-variated Q48-CyPet/YPet spiked with equimolar amounts of non-tagged HTTEx1 mutants with Q48-CyPet/YPet. Lag phases are shifted closer to each other (compare to **(B)**). **(E).** FRET analysis of the aggregation of selected HTTEx1 mutants expressing the physiological Q23. Aggregation of these Q23 mutants were also compared to non-variated Q48-CyPet/YPet control (gray line). **(F)** FRET analysis of non-variated Q48-CyPet/YPet spiked with equimolar amount of HTTEx1 mutants with physiological Q23. Unless stated in the legends, final concentration for each reaction is 3 μM. All results are representative of at least three independent repeats.

Results from the biochemical assays above generally confirmed the role described previously for the flanking domains (Crick et al., 2013; Phan and Schmit, 2020). However, some questions arose when considering the effects of the P1 which we hypothesized could respectively reduce (P+) and increase (ΔP1) the aggregation potential of HTTEx1. We therefore proceeded to further quantify the aggregation kinetics by continuously monitoring the aggregation landscape of HTTEx1 via a FRET based assay. We now assessed the effects of the flanking regions by exploring their behavior also in the presence of a physiological Q23. We generated recombinant GST-tagged HTTEx1 mutants tagged N-terminally to either a CyPet or YPet fluorescent protein, which together constitute a strong FRET pair (Supplementary Figure 1B). When tagged to an aggregation prone protein, an increase of the FRET signal over time becomes a readout of the aggregation kinetics, illustrated in the form of a sigmoidal curve (Figure 3A; Ast et al., 2018a; Scior et al., 2018). When comparing all mutants tagged with CyPet/YPet we were able to replicate the results from our previous biochemical assays (Figure 2). Already 2 h after cleavage of the GST-tag, the P+ mutant (orange line) commenced a fast aggregation process, represented by the steep slope, and reached a plateau within 5 h. In comparison, the non-variated Q48 (gray line), acting as a control, started its aggregation 4 h later. The ΔP1 mutant (purple line) closely followed the control, while the ΔN17 (green line) exhibited the slowest aggregation initiation and fibrillization, reaching a plateau only over a very extended time lapse (Figure 3B). The slopes of these three mutants, Q48, ΔP1 and ΔN17, were also of lesser inclination indicative of milder aggregation kinetics. Unexpectedly, the ΔP mutant (blue line), which all previous biochemical assays confirmed as being highly aggregation prone, did not exhibit a FRET signal over extended time (Supplementary Figure 1E), suggesting its inability to aggregate when fused to CyPet/YPet. The large size of the fluorescent probes immediately after the polyQ domain could in fact inhibit the ability of the polyQ to self-associate and promote aggregation (Holloschi et al., 2014). To test whether we could overcome this structural hindrance and obtain a FRET signal, we titrated increasing amounts of untagged ΔP mutant into the ΔP-CyPet/YPet pair, always maintaining the same final concentration of 3 μM (Figure 3C). Adding only one quarter of untagged ΔP mutant (+1/2x Q48ΔP – turquoise line) to the ΔP-CyPet/YPet mix resulted in a delayed FRET signal compared to the Q48 control (gray line), commencing at approximately 12 hours. Mixing equimolar amounts of tagged and untagged ΔP (+1x Q48ΔP – celeste line) shifted the aggregation initiation to an earlier time point, now matching the control. Further increasing the concentration of untagged ΔP mutant (+2x/+4x Q48ΔP – blue/dark blue lines) resulted in faster aggregation kinetics (Figure 3C). These results suggest that once the polyQ has initiated, uninhibited, its β-structure formation, tagged and untagged moieties can also intercalate into the β-sheet formation, in this case providing a readable FRET signal. It is therefore important to acknowledge that placing any fluorescent probe close to aggregation-prone domains might have profound effects on its aggregation (Riguet et al., 2020).

To further consider the effects of the flanking domains only, and compare simultaneously all mutants, we mixed the non-variated Q48-CyPet/YPet with an equimolar amount of non-tagged soluble HTTEx1 mutant (Figure 3D). As expected, the ΔP mutant accelerated the rate of aggregation (dark blue line), while deleting the anchoring N17 domain (ΔN17) delayed it (green line). Surprisingly, the ΔP1 mutant spike promoted a shift of the lag phase towards an earlier aggregation onset (purple line), before the non-variated Q48. The P+ mutant spike also resulted in a shift (orange line), towards the right and after the non-variated Q48 (gray line), indicating slower aggregation kinetics. These two results are in opposition to the findings of the previous experiment, but not necessarily contradicting: an expanded proline might allow faster self-association in the presence of other expanded prolines, but not as easily when this region is short or missing. Lacking the P1 can bring already formed β-strands into close contact and thus promote further formation of polyQ β-sheets.

To understand whether the contribution of the flanking regions to aggregation is intrinsic to their sequence or whether it is dependent on the presence of an expanded polyQ stretch, we decided to test the ΔP1, P+ and non-variated HTTEx1 mutants containing Q23 in the FRET assay (Figure 3E). Glutamine stretches below the threshold of Q35 are not pathogenic and are not known to aggregate *in vivo*. However, given enough time and at high enough concentrations, shorter stretches of glutamines have been reported to aggregate (Chen and Wolynes, 2017). Our own data confirms this: at very high concentrations (Supplementary Figure 1F), non-variated Q23 has a long lag phase of approximately two weeks (light gray line), before beginning a very slow aggregation phase following the expected sigmoidal kinetics. At the same concentration, the presence of an expanded proline in the Q23P+ mutant (light orange line), drastically reduces the lag phase by a third, and removal of the protective P1 domain in the Q23ΔP1 mutant (pink line), accelerates aggregation six folds. When considering short Q stretches, the proline domain emerges clearly as a regulator of aggregation: removal of P1 might bring the polyQ sufficiently into proximity to trigger a conformational change and initiation of aggregation. Again, elongation of the prolines has a similar outcome as proline-proline interactions also facilitate the association and assembly of the polyQ (Figure 3D). To further dissect the contribution of the flanking domains only, we spiked the non-variated Q48-CyPet/YPet with the HTTEx1 mutants containing Q23 (Figure 3F). Expectedly, the addition of the non-variated Q23 provided the greatest inhibition (light gray line), slowing aggregation and expanding the lag phase. On the opposing side, deletion of the ΔP domain (light blue line), enhanced aggregation almost as if the reaction was containing exclusively constructs with Q48. Elongation or deletion of the P1 (light orange and pink lines, respectively) resulted in a diminished slope inclination, suggesting that the involvement of this domain is somewhat beneficial, as aggregation is slowed, but their role is more complex than a simple delaying function. Interestingly, the deletion of the N17 did not slow aggregation (green line), potentially suggesting that once the initial nucleus is formed, the N17 is not required for further binding; an action that might be promoted by the prolines instead (Figure 3E). Of note, in all FRET experiments curves are normalized to the non-variated Q48; an increase in the efficiency of the FRET signal over 100% can be explained by the positioning and spatial arrangement of the fluorophores, imposed by the presence or absence of HTTEx1 domains themselves.

Overall, our *in vitro* results revealed that expanding the P1 enhanced the aggregation propensities of HTTEx1. Removing the P1 resulted instead in an ambivalent behavior, favoring both an increase and a delay of aggregation, compared to the non-variated HTTEx1 control. While both outcomes are so far contrary to our initial hypothesis, we decided to investigate the role of the flanking domains *in vivo*, in a cellular context, where the PRD might show a clear beneficial involvement against aggregation and toxicity, as promoted by evolution.

### Expanded Prolines Maintain HTTEx1 Soluble *in vivo*

The *in vitro* results obtained so far highlighted that the contribution to aggregation of the PRD, and especially of its P1 domain, is complex. We thus aimed to better understand the impact of the flanking domains on aggregation and consequently correlate these to toxicity in a living animal and during aging by employing the *C. elegans* model organism. The nematode has emerged as a powerful system to study the neurodegenerative disorders and many strains expressing aggregation and disease-related proteins have been generated (Li and Le, 2013; Kim et al., 2016). To our knowledge however, no other *C. elegans* strain exists that expresses the human HTTEx1 throughout the nervous system.

**FIGURE 4:**
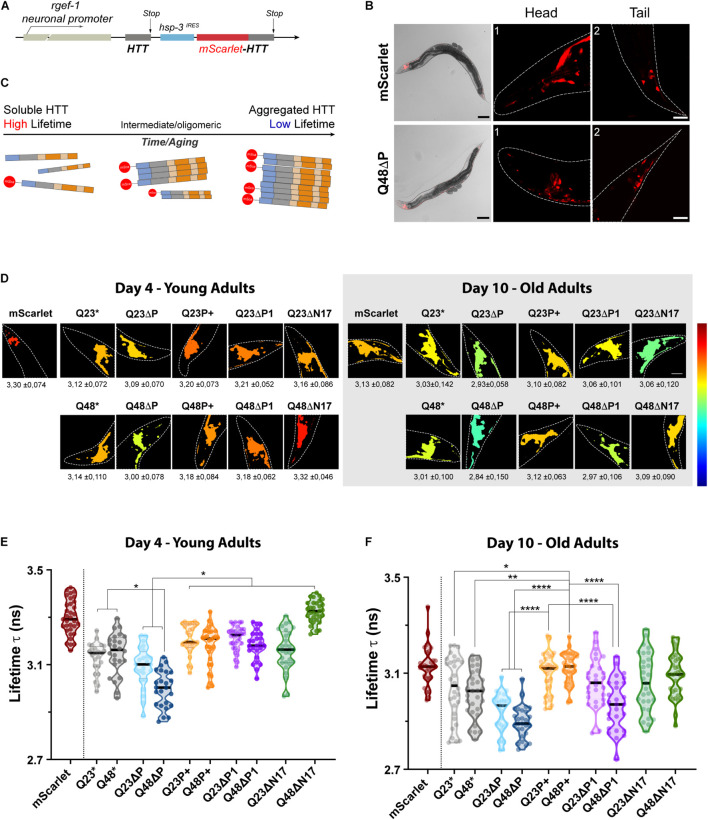
The expanded proline domain maintains HTTEx1 soluble *in vivo*. **(A)** Schematic of the DNA construct used to generate the HTTEx1 *C. elegans* strains. A first moiety of HTTEx1 is transcribed under the control of a constitutively expressed neuronal promoter (*rgef-1)*. A second HTTEx1 moiety fused to the mScarlet is transcribed under the control the *hsp-*3^*IRES*^. **(B)** Representative images of the fluorescent nematode strains. Top panels show the mScarlet control strain, while bottom panels show the Q48ΔP mutant. Whole nematode images, showing the transmission and mScarlet channels, are magnified 100x, scale bar 100 μm; while head (1) and tail (2) neurons, corresponding to the squared highlighted regions, are magnified 630x times, scale bar is 20 μm. **(C)** Graphical representation of the aggregation and consequent lifetime readout of the different constructs by FLIM analysis. Aggregated fractions contain both tagged and untagged HTTEx1 and will record a low fluorescence lifetime τ-value, while soluble moieties of tagged HTTEx1 will maintain a high fluorescence lifetime τ value. **(D)** TCPSC-FLIM pseudo-color images of representative nematode head neurons depicting the average lifetimes for each HTTEx1 *C. elegans* strain, at either day 4 (left) or day 10 (right). Average lifetime τ values ± SD (ns) are stated below each respective image. Dashed lines delimit the head region, magnified ≈1000 folds. High fluorescent lifetimes τ tend towards a red color, while low t tend towards blue, as referenced by the color scale on the left representing 2.3 to 3.5 ns. Lifetime calculations and images were generated with FLIMfit software (v. 5.1.1). **(E,F)** Truncated violin plots representing the average lifetimes recorded from the head neurons of nematode HTTEx1 strains, at day 4 **(E)** and day 10 **(F)** of life. Each circle represents an analyzed nematode. The median is shown by the single black thick line, while quartiles are delimited by above/below dotted lines. Three independent cohorts of at least 10 individual nematodes each were imaged. For statistical significance, a one-way ANOVA followed by Tukey *post hoc* test, assuming a confidence interval of 95%, was performed (*^∗^ = *p* < 0.05; ^∗∗^ = *p* < 0.01*; ^****^ = *p* < 0.0001). For clarity, only selected significance is shown, for a full table of significance refer to Supplementary Table 2.

We generated nematode strains expressing HTTEx1, and all our described mutant forms, with either Q23 or Q48. To follow aggregation, live and *in vivo*, we tagged the HTTEx1 constructs with a nematode codon optimized mScarlet fluorophore. To avoid the steric hindrance of the bulky fluorescent probe on the aggregation kinetics (as seen for the CyPet/YPet constructs *in vitro*; Figure 3C) we employed a strategy that would express the HTTEx1 moiety twice under the control of a single operon (Figure 4A; Gallrein et al., 2021). A non-tagged moiety of HTTEx1 is constitutively expressed in the nervous system of the nematode from early development onwards, under the control of the pan-neuronal *rgef-1* promoter. A second HTTEx1 moiety fused C-terminally to mScarlet, is expressed stochastically and sub-stoichiometrically under the control of the *hsp-3* internal ribosome entry site (*IRES*) (Figure 4B, Supplementary Figure 2A). Over time tagged and non-tagged HTTEx1 proteins are incorporated to form oligomeric and subsequently higher aggregated entities. Importantly, thanks to the transparent properties of the nematode, HTTEx1 can be followed non-invasively and non-destructively in its path of aggregation (Figure 4C, Supplementary Figures 2B–D). To distinguish between the conformational species formed *in vivo*, we employed fluorescence lifetime imaging (FLIM). FLIM has been adapted to distinguish the formation of soluble, oligomeric and aggregated moieties *in vivo* in *C. elegans* (Kaminski Schierle et al., 2011; Gallrein et al., 2021). FLIM measures the fluorescence lifetime (tau, τ), expressed in nanoseconds (ns), of a fluorescent molecule. The lifetime τ is an absolute property intrinsic to the fluorophore itself and varies only when its structural properties or surrounding environment changes. Lifetimes are therefore not steady state intensity measurements and are independent of fluorophore concentrations (Becker, 2012). This feature is particularly important as the expression levels of HTTEx1 are not equal throughout the different strains analyzed and/or exhibit a reduction in levels or suffer instability during aging. Fused to our HTTEx1 mutants, the fluorescent lifetime of mScarlet differs due to its altered conformational setting: when mScarlet is in proximity or encased in the tightly packed environment of an aggregate, the fluorophore undergoes molecular quenching and its apparent τ value diminishes (Figure 4C; Chen et al., 2017). Decrease, or variations, in τ values become a direct proxy readout for HTTEx1 aggregation and the species that populate this transition (Figure 4C).

We therefore set out to investigate and compare the aggregation landscapes of HTTEx1 mutants *in vivo* in our HD *C. elegans* models during aging by measuring the τ values in the head neurons of young, day 4, and old, day 10, nematodes (Figure 4D). Within the cellular environment of a young nematode’s neurons, the average τ of the mScarlet control strain, which expresses only the soluble fluorophore, is 3.3 ns. Compared to this control strain, the average τ of all HTTEx1 strains was significantly lower already at day 4, with the inexplicable exception of ΔN17Q48 strain. Noticeably, the τ values of ΔP mutants were the lowest recorded for both the Q23ΔP and especially the Q48ΔP expressing strains, which dropped as low as 2.85 ns, demonstrating strong aggregation *in vivo* (Figure 4E, Supplementary Table 2). These results are in agreement with previous data (Dehay and Bertolotti, 2006; Gruber et al., 2018) and highlight the protective role of the PRD, which, once removed, exacerbates the aggregation propensity of HTTEx1, even when a short non-pathogenic polyQ stretch is present. P+, ΔP1 and ΔN17 HTTEx1 mutant strains all exhibited significantly higher τ values compared to the ΔP mutants, almost all above 3 ns and up to 3.4 ns, indicating that a larger part of the PRD is required to maintain HTTEx1 soluble. Interestingly, the non-variated HTTEx1 exhibited a τ value significantly higher only compared to the Q48ΔP mutant, raising ambiguity regarding the presence and role of the P1 region in the aggregation propensities of HTTEx1, at early life stages (Figure 4E, Supplementary Table 2). For each of the other HTTEx1 mutants, the average τ values were similar despite the presence of physiological Q23 or expanded Q48, confirming that in young nematodes, the organism is still capable of suppressing aggregation (Labbadia and Morimoto, 2015).

In old nematodes at day 10, we noticed immediately a significant reduction of τ for all HTTEx1 mutants, including the mScarlet control, but excluding the Q48P+ strain (Figure 4F). The τ of Q48P+ remained elevated, with an average value of 3.18 ns and 3.12 ns at day 4 and day 10 respectively, indicating that the expanded proline region maintains soluble HTTEx1 containing a pathologic stretch of Q48 during aging (Supplementary Figure 3A; Supplementary Table 2). For all mutants, including the control, an increase in aggregation and consequently decrease in τ is expected as the result of natural aging, changes in cellular environment, and collapse of the proteostasis network (Ben-Zvi et al., 2009). Importantly, there was a widening of τ values among the tested cohorts in almost all mutants, indicating the presence of a more variated, and possibly polymorphic, population of soluble, oligomeric and aggregated species formed during aging (Figure 4F). This observation was particularly true for both ΔP1 mutants, containing either Q23 or Q48, in which deletion of the P1 region resulted in a much broader and lower set of average τ values during aging, ranging between a high of 3.27 ns and a low of 2.74 ns. Here, different populations of oligomeric species were clearly visible, illustrated by the repeating bulging areas of the violin plots. Nonetheless, the beneficial effects of the P1 were highlighted by the higher τ maintained in the strains expressing an expanded proline, in both Q23 and Q48. During aging, Q23P+ and Q48P+ strains exhibited significantly higher τ values, with an average above 3.10 ns and similar to the mScarlet control, and with less species variation, suggesting an overall ability to maintain HTTEx1 soluble. In contrast, the ΔP mutants again recorded the overall lowest τ, indicating that aggregation worsens further over aging and especially when the protective PRD domain is missing, illustrated by a reduction in average τ values from above 3 ns to a low of 2.84 ns. Our FLIM results emphasize the modulations imposed by the flanking regions, and specifically the importance of the P1, and generally the PRD, in delaying aggregation and maintaining the solubility of HTTEx1 in living and aging *C. elegans*.

### Expanded Prolines Promote Beneficial Behavior *in vivo* Only for the Pathogenic Q Stretch

**FIGURE 5:**
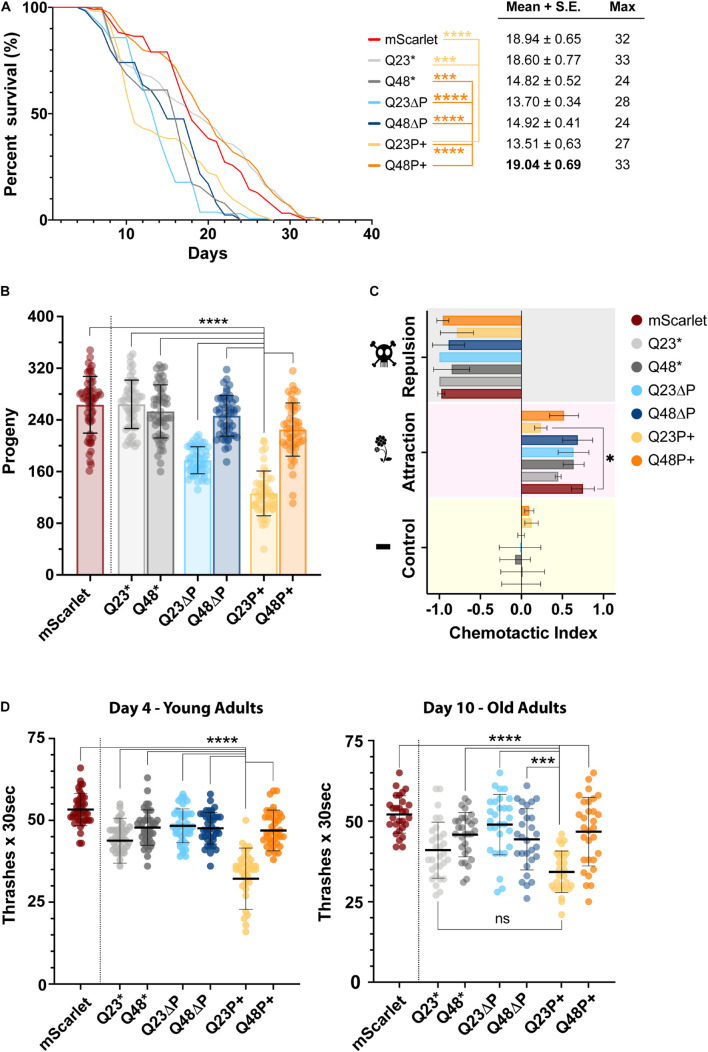
The expanded proline is beneficial *in vivo* in the presence of pathogenic, but not physiological polyQ tracts. **(A)** Lifespan assay, illustrated with a Kaplan-Mayer plot, of selected *C. elegans* HTTEx1 mutant strains alongside the mScarlet control strain. Three independent repeats were performed of at least 120 nematodes each. On the right are shown the mean and standard error, and the maximum lifespan values for each strain. For significance a log-rank test was performed (^∗∗∗^ p < 0.000001; ^****^ = p < 0.0000001). Only selected values of significance are shown, for a full table of significance refer to Supplementary Table 3. **(B)** Scatter dot plot with bar of the progeny assay performed for selected *C. elegans* HTTEx1 strains and mScarlet control. The average number of offsprings recorded from three independent repeats, of 20 nematodes each, is shown as mean ± SD. Significance is calculated performing a one-way ANOVA followed by Tukey’s *post hoc* test and assuming 95% confidence interval (^****^ = p < 0.0001). **(C)** Grouped bar plot of chemotactic assay performed on selected *C. elegans* HTTEx1 mutant strains alongside the mScarlet control strain. Shown is the mean ± SD of three independent repeats. The chemotaxis index is calculated between the whole nematode plate cohort and the nematodes present on the test substance quadrant, either an attractant (1% benzaldehyde), a repellent (50% benzaldehyde) or a control substance (EtOH). Significance was tested with a one-way ANOVA followed by Tukey’s *post hoc* test and assuming 95% confidence interval (^∗^ = *p < 0.05*). **(D)** Scatter dot plot of the thrashing assay performed on selected *C. elegans* HTTEx1 strains and mScarlet control strain, on young day 4 nematodes and on old day 10 nematode. The average number of thrashes performed in M9 liquid medium every 30 s is shown as mean ± SD. Between 30-45 nematodes per strain were analyzed in three independent repeats. For significance, a one-way ANOVA followed by Tukey *post hoc* test assuming 95% confidence interval was performed (*^∗∗∗^ = p < 0.001*; ^****^ = p < 0.0001). For clarity, only selected significance results are shown for figures **(B)** and **(D)**, for a full list of significance refer to Supplementary Table 4.

The polyQ flanking regions of HTTEx1 have a role in aggregation that has barely been studied or appreciated *in vivo* (Shen et al., 2016). Our FLIM measurements highlight the ability of these domains to directly influence aggregation, positively and negatively, by either promoting the solubility or advancing the oligomerization and aggregation of HTTEx1. With its expanded polyQ domain, HTTEx1 is a toxic protein either by loss of function or gain of toxic function (Zheng and Diamond, 2012). The exact nature of the toxic species is still highly debated, and several lines of evidence demonstrate all structures, soluble, oligomer or aggregated protein, as potential culprits of neuronal cytotoxicity (Arrasate and Finkbeiner, 2012). With knowledge from FLIM measurements of the nature of the mutants’ conformation (Figures 4E,F), we investigated HTTEx1’s toxicity in *C. elegans*. We characterized our models and the damage inflicted by each mutant on the physiology of the nematode by performing a set of phenotypic assays (Figure 5 and Supplementary Tables 3, 4). For clarity, we focused on the ΔP and P+ strains, and their controls, but results and analyses for all HTTEx1 mutant strains can be found in the Supplementary material (Supplementary Figures 3B–E and Supplementary Tables 3, 4).

We first analyzed the impact of mutant HTTEx1 on the lifespan of the nematode by performing a survival assay (Figure 5A), an established method to assess the toxicity of amyloidogenic proteins in models of neurodegenerative diseases. It quickly emerged that both Q23ΔP and Q48ΔP strains (light blue and dark blue lines respectably), which are devoid of the protective PRD and exhibited high aggregation load in the FLIM measurements (Figure 4F), were quickly dying. Surprisingly, the Q23P+ (light yellow line) nematodes also exhibited a reduced half-life and perished significantly faster than the mScarlet control (red line). Conversely, the Q48P+ strain (orange line) exhibited a survival rate close to that of mScarlet control and to the non-variated Q23 strains (light gray line), indicating that an expanded proline adjacent to an expanded polyQ is beneficial in promoting survival. ΔP1 and ΔN17 strains also exhibited long lifespans, again highlighting the importance of possessing a PRD, either in its entirety or with minor modifications (Veldman et al., 2015) (Supplementary Figure 3B). The lifespan is useful to assess the general impact of HTTEx1 on the nematode. To assess early adverse effects on the fitness of the organism, we performed a fecundity assay (Figure 5B, Supplementary Figure 3C). After counting the progeny of each HTTEx1 mutant strain, it became apparent that the Q23P+ strain (light orange column) was severely impaired in its reproduction capacities, by more than half, compared to all other mutant strains, while a less pronounced reduction in egg laying was also noticeable for the Q23ΔP nematodes (light blue column). Analysis of several independently generated Q23ΔP lines also reported a decreased progeny count, suggesting that the impaired fecundity is not due to damage provoked by integration into genes specifically important for reproduction. Such drastic reduction in offspring number for the Q23P+ strain pointed to a severe, and unexpected, impairment imposed by the expanded proline on the health of the organism from early stages. To characterize the neurodegenerative component of our HD model strains, we then assessed neuronal function by subjecting the nematodes to a chemotactic assay (Figure 5C, Supplementary Figure 3D): fit nematodes move away from a volatile repulsive substance, move towards an attractant, while having no preference for a neutral stimulus. Selected nematode strains responded as expected to the repulsive, and potentially harmful substance, and to the control. In response to the attractant, a mixture of behaviors was observed: again, the Q23P+ nematodes were impaired compared to controls, further demonstrating the extensive detrimental potential of this HTTEx1 mutant on the fitness of the nematode (Figure 5C). Interestingly, the ΔN17 mutants performed poorly overall, possibly due to their compromised locomotion, a feature reported by other models lacking the N17 domain (Gu et al., 2015). We cannot however explain the complete inability of ΔP1 strains to detect attractive odorants (Supplementary Figure 3D). Finally, to assess the stability or degeneration of muscle-neuron connectivity, we performed a motility assay (Figure 5D). We counted the number of thrashes performed in liquid for each HTTEx1 mutant strain at both day 4 and day 10 of the nematodes’ life. The Q23P+ strain was unable to perform more than 35 thrashes every 30 s, less than half compared to the average of most other HTTEx1 mutant strains, including the Q48P+ (Figure 5D). During aging, the same significance pattern emerged, albeit all strains experiencing a general diminishing in the average number of thrashes. Again, the motor impairment of the ΔN17 mutants is reflected in their decreased thrashing ability, which worsens significantly over time (Supplementary Figure 3E).

Overall, the phenotypic characterization of the nematode HTTEx1 strains revealed a complex picture of behaviors imposed by the presence or absence of the flanking domains, or indeed by the polyQ itself. The lifespan assay confirms the toxic effects of aggregation load on survival, in accordance with the FLIM data. Instead, the effect of aggregates, or even oligomeric and monomeric species, is much more nuanced in other nematode behaviors tested. Further assays are warranted to understand the biological relevance and impact of HTTEx1 in the nervous system of *C. elegans* to fully appreciate the subtle difference imposed by all HTTEx1 flanking regions.

## Discussion

Huntington’s disease is caused by an expansion in the polyQ stretch of HTTEx1. Although this genetic cause of the disease is undisputed, the actual involvement of the protein’s polyQ region in pathology is being scrutinized. In this study, we investigated and dissected the contribution of the domains flanking the polyQ stretch in HTTEx1 and elucidated the specific contribution of the first pure repeat of prolines, the P1, immediately adjacent to the C-terminal of the polyQ. We hypothesized that the P1 has been expanding in mammals to counteract the concomitant expansion of the polyQ, thus allowing HTTEx1 to maintain, or even improve its function, rather than promote pathology. Unlike previous studies that employed only fragments or unnatural mutations of HTTEx1, we analyzed the N17 and PRD, in the context of both physiological and pathological polyQ expansions, and their singular involvement in aggregation *in vitro* and *in vivo.* Our *in vitro* experiments employed both fusion constructs and untagged HTTEx1 recombinant proteins, to prevent altered ultrastructural and biochemical properties imposed by the fluorescent tags (Vieweg et al., 2016). Similarly, in our *C. elegans* models we circumvented the potential issue imposed by steric hinderance of the fluorophore and studied HTTEx1 aggregation and toxicity in a relevant cellular and organismal context, without excluding any contributions from PTMs, tissue crosstalk, interaction partners and aging. Our results showcase some contradictive behavior between *in vivo vs in vitro* experiments (Supplementary Figure 4; Lashuel, 2021), but nonetheless emphasize a clear involvement of flanking domains in defining the aggregation landscape of polyQ proteins. Specifically, our study in living animals confirmed the PRD as the protective entity of HTTEx1 and the P1 as the evolutionary addition to expanding polyQ with the purpose of maintaining HTTEx1 function whilst avoiding pathology.

Similar to previous studies we found that the PRD exhibited a protective function and, once removed, the remaining N17-polyQ fragment exacerbated the aggregation propensity of HTTEx1 (Bhattacharyya et al., 2006; Dehay and Bertolotti, 2006; Darnell et al., 2007). All biochemical assays confirmed that Q48ΔP aggregated with fast kinetics into large structures that were fully SDS-insoluble (Figures 2B,F). Q48ΔP mutants had a detrimental effect also on the aggregation kinetics of non-variated Q48, when mixed, facilitating a faster and earlier aggregation (Figure 3D). An increase in surface availability between the polyQ stretches of the non-variated Q48 and of Q48ΔP might occur once the hinderance imposed by the PRD helix, or indeed the fluorophore, was removed (Figures 3C,D). This shift in aggregation kinetics of non-variated Q48 occurred even when the PRD was missing in the context of Q23, further highlighting the protective role of the PRD, independently of polyQ length (Figure 3E). Absence of the PRD translates into a loss of the dynamic PPII helix (Darnell et al., 2009): the remaining polyQ, regardless of its Q number, is possibly facilitated into transitioning from a helical structure into an aggregation-prone β-sheet (Kim et al., 2009; Bugg et al., 2012). While our *in vitro* results are in agreement with previous studies, we further confirmed the behavior of the PRD *in vivo* in a living and aging organism. FLIM measurements demonstrated that removing the PRD resulted in the presence of aggregated species already in young nematodes, which increased with aging for both polyQ expansions (Figures 4E,F). When correlating aggregation load to toxicity, lifespans of these ΔP nematode strains were significantly decreased compared to controls (Figure 5A). However, their overall fitness, measured by chemotaxis, motility and fecundity assays, did not appear impaired, except for the fertility of the Q23ΔP strain (Figure 5B). These results suggest that aggregates themselves might not actively disrupt important processes, nor are they toxic from their inception. Instead, over time aggregates could remove from the cell the necessary resources required for proper cell functioning, ultimately leading to the collapse and death of the organism, through for example sequestration of chaperones or space utilization (Kaganovich et al., 2008; Olzscha et al., 2011; Arrasate and Finkbeiner, 2012). Previous studies reported that large fibrillar aggregates intercalate with membranous compartments, for example of the endoplasmic reticulum, with noxious consequences on cell integrity (Kirstein et al., 2015; Bäuerlein et al., 2017). Furthermore, cleavage or disaggregation of large inclusion into smaller fibrils also contributed to seeding effects and potential non cell autonomous propagation of toxic species (Tipping et al., 2015; Tittelmeier et al., 2020). While removal of the PRD is a trigger of aggregation and aggregates have negative consequences on the cell’s homeostasis, the monomeric ΔP could also be involved in promoting toxicity. The PRD itself was found to be the site of important binding interactions, as proline residues specifically engage partner proteins containing SH3 and WW domains. Disruption of these interactions resulted in loss of non-reception signaling, degradation capabilities and mRNA processing, contributing to HD pathology (Liu et al., 1997; Faber et al., 1998). In a previously established mouse model of HD that lacked the PRD no dysfunctional phenotype or developmental impairment compared to wild type was observed and only a late life loss of memory was reported (Neveklovska et al., 2012). Notably, full length HTT was used in this study, and the presence of other HTT domains might compensate the loss of the PRD and its many interactions. Indeed the precise role of the PRD appears complex and its subtle function in mammals with higher cognitive capabilities is still hard to fully appreciate.

Huntingtin is a conserved protein throughout species, with orthologs emerging already in amoebas. The proline rich region, and specifically P1, is a recent evolutionary adjunction present exclusively in mammals: its increasing length correlating to the apparent cognitive and brain expansion (Zuccato and Cattaneo, 2016). The presence of a C-terminal polyP stretch of only 5 amino acids is already sufficient in delaying aggregation (Bhattacharyya et al., 2006). Repeats of pure prolines are not placed randomly within protein sequences and serve a definite function especially when flanking Q stretches (Ramazzotti et al., 2012), and indeed homo-repeats of any kind permit specialized biological tasks (Jorda and Kajava, 2010). Expanding the P1 region, from 11P to 22P, resulted in an increased aggregation potential *in vitro* (Figures 2, 3), possibly due to the extended contact area newly created by the large proline stretch, which are able to engage in homotypic binding, as occurs in other proline rich proteins (Vazquez-Portalatin et al., 2020). The same increased aggregation potential was promoted by 22P when flanking a short Q23 stretch, suggesting that the homo-proline association proceeds the polyQ association and is sufficient to enhance the polyQ-mediated effects (Figures 3E,F). Q48P+ aggregates were however SDS-soluble, in contrast to the ΔP mutant, suggesting that the presence of prolines might produce HTTEx1 aggregates that are more fragile and frangible in nature (Shen et al., 2016). PolyP-induced binding and fibril formation might represent a less stable interaction than that produced by the polar zipper of the polyQ, as the transition into a β-structure might be avoided or delayed. In the packed environment of the cell, however, the highly dynamic expanded P22 cannot self-associate as freely as *in vitro* and might become a better target for interacting partners. Furthermore, the helical nature of the polyP could prevent the formation of an aggregate-inducing β-structure by conformationally propagating itself into the polyQ, maintaining the high energy barrier required to prevent polyQ transition into a stable β-sheet (Hoop et al., 2016; Urbanek et al., 2020a, b). Indeed, HTTEx1 with an expanded polyP stretch is maintained soluble *in vivo*, regardless of polyQ size, as demonstrated by the high fluorescence lifetime τ recorded for both Q23P+ and Q48P+ (Figure 4D). The high solubility of HTTEx1 translated into beneficial effects for the fitness and survival of the nematode (Figure 5), but surprisingly, only for the Q48P+. This result was in agreement with our hypothesis that expanding prolines counteracted the effects of expanded glutamines. Expanded prolines however were extremely deleterious in the presence of short non-pathogenic polyQ stretches (Figure 5). Counterintuitively, HTTEx1 containing a short polyQ flanked by a long polyP conferred higher toxicity to an animal than HTTEx1 with an expansion of both polyQ and polyP. These results point to the flanking domains as joint regulators of toxicity *in vivo* rather than the mutant polyQ alone.

**FIGURE 6:**
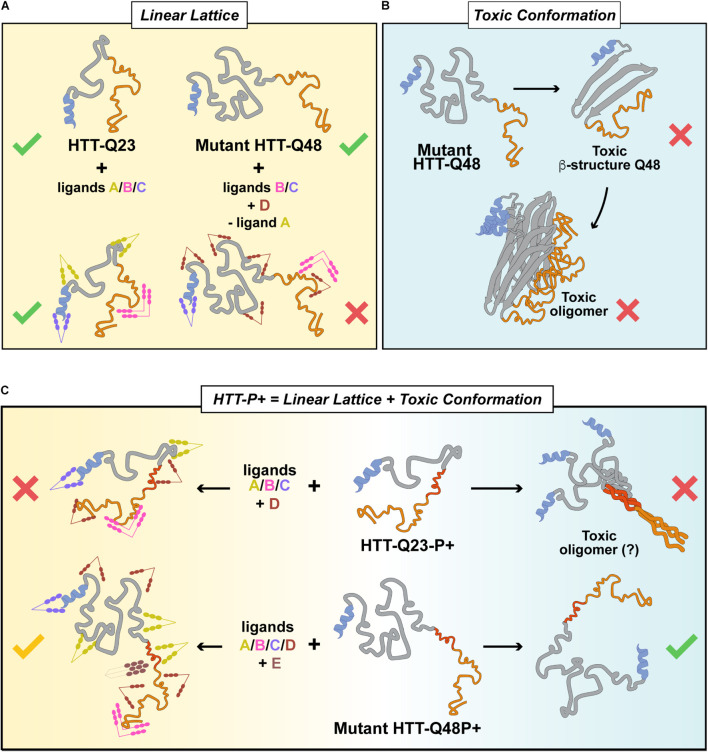
HTTEx1 P+ behaves both according to the linear lattice and the toxic conformant models. **(A)** The *linear lattice* model of HTTEx1 postulates that mutant HTTEx1 itself is not intrinsically toxic. In the presence of Q48, but not Q23, the loss of important interactions partners or the addition of novel ones, renders the monomeric HTTEx1 toxic via a gain of toxic function. **(B)** Conversely, the *toxic conformant* model postulates that mutant HTTEx1Q48 is toxic as the expanded polyQ is capable of folding into a new β-structure that favors assembly into oligomeric species which are intrinsically toxic. **(C)** In the presence of an expanded proline, both models might be correct: Q23P+ is toxic *in vivo* as it might both increase the surface availability for ligands, and/or assume a different conformation, arising from a structural rearrangement imposed by the long polyP onto the short polyQ. For Q48-P+, the transition into toxic oligomers might be prevented by the long polyP hindering the structural rearrangement of the expanded polyQ, thus diminishing toxicity. Overall however, the interaction network of HTTEx1-P+ might also favor an increased amount of ligand binding, some of which might result in either beneficial or deleterious effects.

In what form and how HTTEx1 causes pathology is still unknown, as both a detailed structure (Guo et al., 2018) and a thorough characterization of its function are incomplete. Theoretical and experimental evidence based on biological data and *in silico* simulations have produced contradicting and contrasting models. The linear lattice model (Figure 6A) suggested that monomeric HTTEx1, either possessing a physiologic or a pathogenic polyQ stretch, maintains the same overall structure *in vitro* oscillating between a random coil or α-helices (Bennett et al., 2002; Li et al., 2007; Bravo-Arredondo et al., 2018). Toxicity arises as expanded Qs allow for a greater number of binding sites due to a larger polyQ surface, which in turn stimulates ligand interactions (Warner et al., 2017; Newcombe et al., 2018). These avidity effects cause higher affinity interactions and result in altered binding with partner proteins or other polyQ domains, leading to aggregation and neurotoxicity. However, heterotypic interactions, some of which might push mutant HTTEx1 into a gain of toxic function, could also present with protective effects, depending on the nature of the ligand (Posey et al., 2018; Ceccon et al., 2020). In contrast to this hypothesis, the ‘emergent conformation’ model, also known as ‘structural toxic threshold’ (Figure 6B), proposes that HTTEx1 assumes a length-dependent toxic conformation by transitioning from monomers into dimers, trimers and tetramers, into larger still diffusible small oligomers, and down the amyloid formation pathway. It is still debated, which of these conformational states represent the toxic species, but HTTEx1 needs to adopt a β-hairpin structure to promote cytotoxicity (Sahoo et al., 2016; Wetzel, 2020). Breakage of this structure by placing a mutation within the polyQ, or the N17, abrogated the formation of amyloids *in vitro* (Thakur and Wetzel, 2002; Atwal et al., 2007). HTTEx1 mutants that instead transitioned quickly into β-structures promoted the formation of neurotoxic oligomeric species, even with non-pathological Q stretches (Truant et al., 2008; Drombosky et al., 2018). Results from our investigation suggest that both models have merit and taken together can fully explain HTTEx1 toxicity, especially in the presence of an expanded P1 (Figure 6C). In accordance with the lattice model, for which monomeric species would be responsible for toxicity (Nagai et al., 2007), expanded prolines would allow for larger surface binding interaction between HTTEx1 moieties and both aggregation-enhancing and aggregation-delaying ligands. First, our *in vitro* data confirmed the ability of the dynamic proline stretch to form soluble fibrillar structures, which translates into an overall high binding capacity. Our FLIM data further suggested that the conformational species present in the nematode are oligomeric, although the exact size is unknown, as single molecule characterization is still outside of the measurement’s sensitivity. These oligomeric species might remain inert by binding to aggregation-delaying interactors. Alternatively, due to the structural rearrangements imposed by the flanking domains, and especially by the expanded polyP adopting a PPII helix (Blanch et al., 2000), HTTEx1 could transition into a novel fold, of yet undefined structure. The influence newly imposed by the expanded polyP onto the polyQ differ in relation to the polyQ length itself. An altered, now toxic, conformational state is imposed by expanded prolines onto short Qs and would explain how this Q23P+ construct causes deleterious effects in the nematode’s fitness and survival. Conversely, the same polyP expansion would structurally propagate itself onto the expanded polyQ with a different outcome. The resulting structure proved to be a less toxic conformation, evident by the beneficial effects on fitness and survival in the Q48+ nematode strain. Our results in this scenario now align with the emergent conformation model (Miller et al., 2011), which supports the rearrangement of HTTEx1 into β-hairpin structures of oligomeric nature as being the toxic culprits. It therefore emerges how the sequence context is much more important for modulating toxicity than the polyQ alone (Lakhani et al., 2010). What structure does the expanded P1 impose on the polyQ, what novel partners might it recruit autonomously or in cooperation with varying polyQ, and what is its role within the whole PRD, are all questions that need to be addressed. Investigation of the flanking regions of other polyQ disease proteins, for example the ataxins, and their effects on the structure/toxicity relationship, will further aid in understanding the importance of the protein context and help uncover how variability in neuronal vulnerability or pathological processes reside in the interplay between the polyQ and its flanking domains. For HD the role of the flanking domains in functionality and toxicity *in vivo* is slowly being unraveled. Focusing on the evolutionary newer PRD could also answer why the polyQ is expanding in humans and how natural selection pressure is working to prevent this functional mutation from causing a devastating disease.

## Data Availability Statement

The original contributions presented in the study are included in the article/Supplementary Material, further inquiries can be directed to the corresponding author/s.

## Author Contributions

MLP and JK conceptualized the study, designed the experiments, and wrote the manuscript. MLP and ML performed the experiments. AM contributed to the imaging analysis. GSKS and JK provided supervision. All authors contributed to the article and approved the submitted version.

## Conflict of Interest

This study received funding from Infinitus China Ltd. The funder was not involved in the study design, collection, analysis, interpretation of data, the writing of this article or the decision to submit it for publication. The authors declare that the research was conducted in the absence of any commercial or financial relationships that could be construed as a potential conflict of interest.

## Publisher’s Note

All claims expressed in this article are solely those of the authors and do not necessarily represent those of their affiliated organizations, or those of the publisher, the editors and the reviewers. Any product that may be evaluated in this article, or claim that may be made by its manufacturer, is not guaranteed or endorsed by the publisher.
