# Supplementary material for: An Expanded Polyproline Domain Maintains Mutant Huntingtin Soluble in vivo and During Aging
Source: Front Mol Neurosci. 2021 Oct 15;14:721749. doi: 10.3389/fnmol.2021.721749 (PMC8554126; doi:10.3389/fnmol.2021.721749)
Supplement: Supplementary file 1 [file Data_Sheet_1.docx]

Supplementary Material

# Supplementary Methods

**Western blot analysis of *C. elegans* strains**

For Western blot analysis, nematodes were lysed with a crude boiling method: 50-100 nematodes were collected in low protein binding tubes and washed 5x with M9 buffer. Supernatant was almost completely removed and 4xLB was added to the nematode pellet. Samples were briefly vortexed, spun and then boiled for 15 min at 100°C and 1400 rpm shaking. Samples were subjected to 10% SDS-PAGE gels in Tris-Glycine buffer. For all strains except ∆N17, proteins were transferred to a nitrocellulose membrane via semi-dry blotting (Trans-Blot Turbo System, BioRad). For ∆N17 strains, proteins were transferred to a PVDF membrane via wet blotting at 90 V for 2 hrs. Membranes were blocked in 5% skim milk in TBS-T for 1 hour at RT and then probed with primary anti-HTT EPR5526 (1:1000; Abcam) for controls, ΔP, P+ and ∆P1 strains, or anti-HTT 3B5H10 (1:1000; Merck) antibody for ΔN17 strains and controls. For secondary antibody, HRP-conjugates anti-rabbit and anti-mouse antibodies (1:10,000, #31460 and #31444, Thermo Fisher) were applied. Blots were imaged on a Lumi-Imager F1 system and employing LumiAnalyst software (Boehringer Mannheim GmbH).

**Immunohistochemistry of *C. elegans***

Immunohistochemistry of HTT strains was performed as previously described (Nonet et al., 1993), following the collagenase IV method. Treated nematodes were incubated for 24 hrs with primary antibodies against EPR5526 (1:100; EPR5526, Abcam) for control, ΔP, P+ and ∆P1 strains, and 5TF1-3B5H10 (1:100; Merck) for controls and ΔN17 strains and probed with Alexa Fluor 647 anti-rabbit or anti-mouse secondary antibodies (1:10,000, ab150075 and ab150115, Abcam). Nematodes were embedded in VectaShield media containing DAPI to stain cell nuclei (Vector Laboratories) and imaged on a Leica SP8 with HC PL APO CS2 100×/1.40 NA oil objective (Leica Microsystems) and with a supercontinuum white light laser (NKT Photonics) with excitations/emissions (ex/em) set at 405/440nm, 561/590nm and 637/750nm for DAPI, mScarlet and HTT, respectively.

**References**

Nonet, M. L., Grundahl, K., Meyer, B. J., and Rand, J. B. (1993). Synaptic function is impaired but not eliminated in C. elegans mutants lacking synaptotagmin. *Cell* 73, 1291–1305. doi:10.1016/0092-8674(93)90357-V.

# Supplementary Figures and Tables

**Supplementary Table S1.**

***C. elegans* Huntington´s disease models**

| ***C. elegans Strain*** | ***Genotype*** |
| --- | --- |
| **mScarlet** | *ppD95_rgef-1::SM-IRES-mSCA-NP::unc54-3’UTR* |
| **Q23*** | *pPD_95-rgef-1::HTTQ23*-IRES-mSCA:: HTTQ23*-unc54-3’UTR* |
| **Q48*** | *pPD_95-rgef-1::HTTQ48*-IRES-mSCA:: HTTQ48*-unc54-3’UTR* |
| **Q23∆P** | *pPD_95-rgef-1::HTTQ23∆P-IRES-mSCA:: HTTQ23∆P-unc54-3’UTR* |
| **Q48∆P** | *pPD_95-rgef-1::HTTQ48∆P-IRES-mSCA:: HTTQ48∆P-unc54-3’UTR* |
| **Q23P+** | *pPD_95-rgef-1::HTTQ23P+-IRES-mSCA:: HTTQ23P+-unc54-3’UTR* |
| **Q48P+** | *pPD_95-rgef-1::HTTQ48P+-IRES-mSCA:: HTTQ48P+-unc54-3’UTR* |
| **Q23∆P1** | *pPD_95-rgef-1::HTTQ23∆P1-IRES-mSCA:: HTTQ23∆P1-unc54-3’UTR* |
| **Q48∆P1** | *pPD_95-rgef-1::HTTQ48∆P1-IRES-mSCA:: HTTQ48∆P1-unc54-3’UTR* |
| **Q23∆N17** | *pPD_95-rgef-1::HTTQ23∆N17-IRES-mSCA:: HTTQ23∆N17-unc54-3’UTR* |
| **Q48∆N17** | *pPD_95-rgef-1::HTTQ48∆N17-IRES-mSCA:: HTTQ48∆N17-unc54-3’UTR* |

List of genotypes of *C. elegans* expressing HTTEx1 mutants. (*) denominates non-variated HTTEx1. Each HTTEx1 moiety terminates with a stop codon.

**Supplementary Tables S2 – S3 – S4**

Please refer to the Excel sheets for Supplementary Tables S2, S3 and S4.

**Supplementary Figure S1.**

**
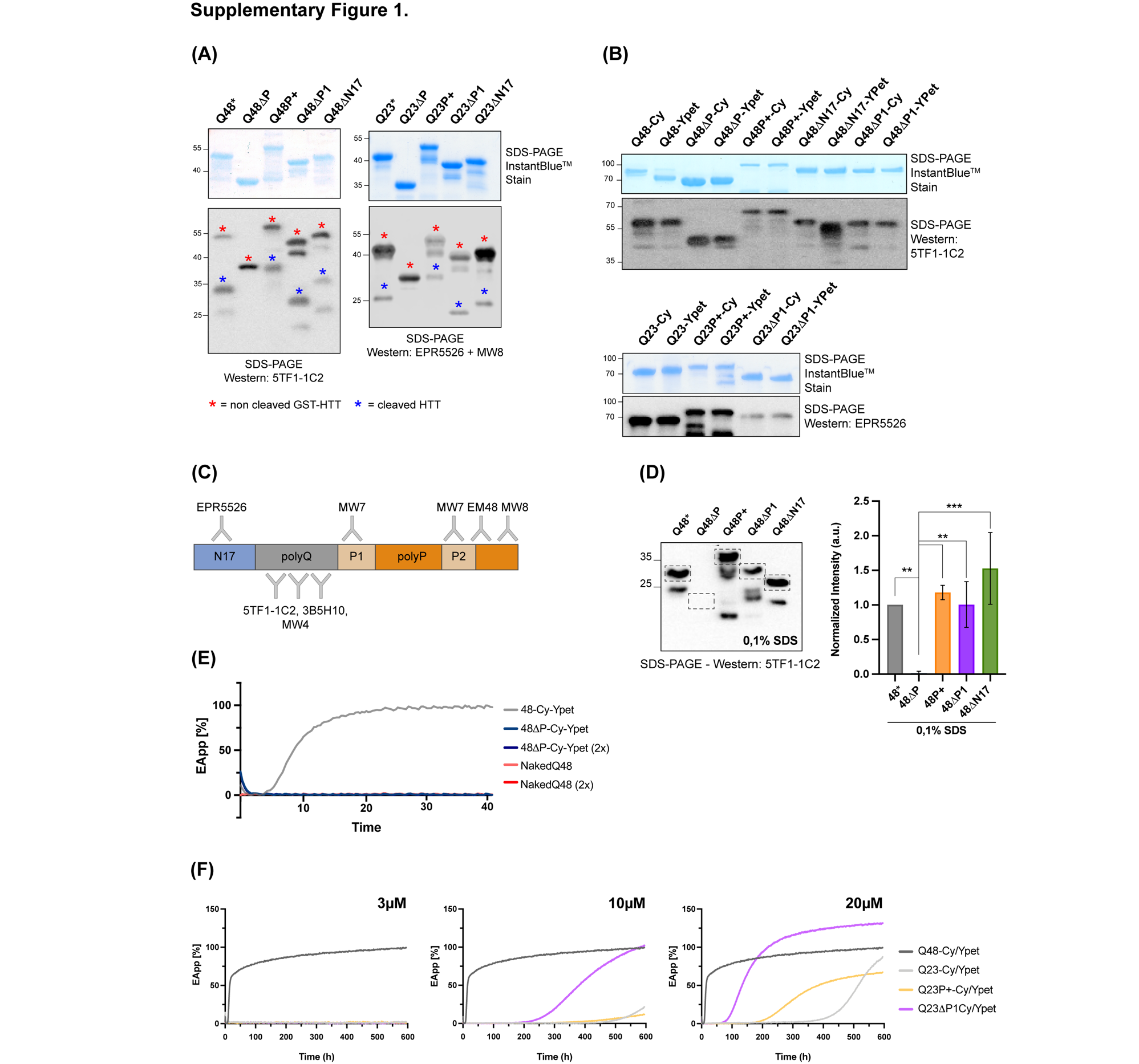
**

**A.** SDS-PAGE of GST-tagged purified Q48 (left panels) and Q23 HTT mutant proteins (right panels). Top panels show non-cleaved proteins, stained with InstantBlue Coomassie stain. Bottom panels show Western blots of HTTEx1 constructs 4 hours after addition of PP. Blotting was performed with the polyQ specific 5TF1-1C2 antibody for Q48 constructs and with both the EPR5526 and MW8 antibodies for Q23 constructs. Asterisks represent the un-cleaved (top) and cleaved HTT moieties, but likely truncated species were also detected by the antibodies. **B.** SDS-PAGE of GST- and CyPet- or YPet- tagged purified HTT proteins. Top panels represent Q48 constructs, lower panels represent Q23 constructs. For Q23 only the ∆P1, P+ and non variated exon 1 constructs were analyzed. InstantBlue stained gels represent HTTEx1 mutant proteins in the absence of PP, while Western blots depict HTTEx1 mutants cleaved by PP for 4 hours. Q48 is blotted against the 5TF1-1C2 antibody, while Q23 constructs are blotted against EPR5526 antibody. **C.** Schematic of HTTEx1 and the epitopes recognized by the antibodies utilized in this study. EPR5526 binds the N17 domain, 3B5H10, MW4 and 5TF1-1C2 bind the expanded glutamine stretch, MW7 binds both polyproline regions, EM48 and MW8 bind the terminal amino acids of the exon 1. No single antibody, either used or researched in the literature, was able to recognize all HTTEx1 mutants at the same time. **D.** SDS stability assay performed by Western blot analysis and quantification. Blots represent the amount of soluble HTTEx1 present upon treatment with 1% SDS after 24 hours of aggregation. Immunoblot stained for the polyQ-specific 5TF1-1C2 antibody. Bands utilized for quantification are boxed; lower bands represent truncated species and were not quantified. Shown here is one of three independent repeats. **E.** FRET assay representing the aggregation of the Q48∆P-CyPet/YPet (blue lines) or pure nakedQ48-CyPet/YPet pair (red lines), compared to Q48-CyPet/YPet control (gray line). Neither Q48∆P nor a nakedQ48, a pure polyQ construct devoid of flanking regions, aggregated, over extended time or at double concentration, due to the steric hindrance imposed by the fluorophores, which prevent the polyQs from forming a polar zipper. **E.** FRET assay representing the aggregation kinetics of Q23 mutant at increasing concentration. At the same 3µM concentration of the Q48 control (dark gray line), no signal is recorded after 600 hours for any of the Q23 mutants. At 10µM concentration the Q23∆P1 mutant (pink line) stats aggregating at approx. 250 hours, while only a beginning of aggregation is visible for Q23* (light gray line) and Q23P+ (light orange line). At further increasing concentration of 20µM (and 40µM – Fig. 3E), full kinetics of aggregation are finally visible.

**Supplementary Figure S2.**

**
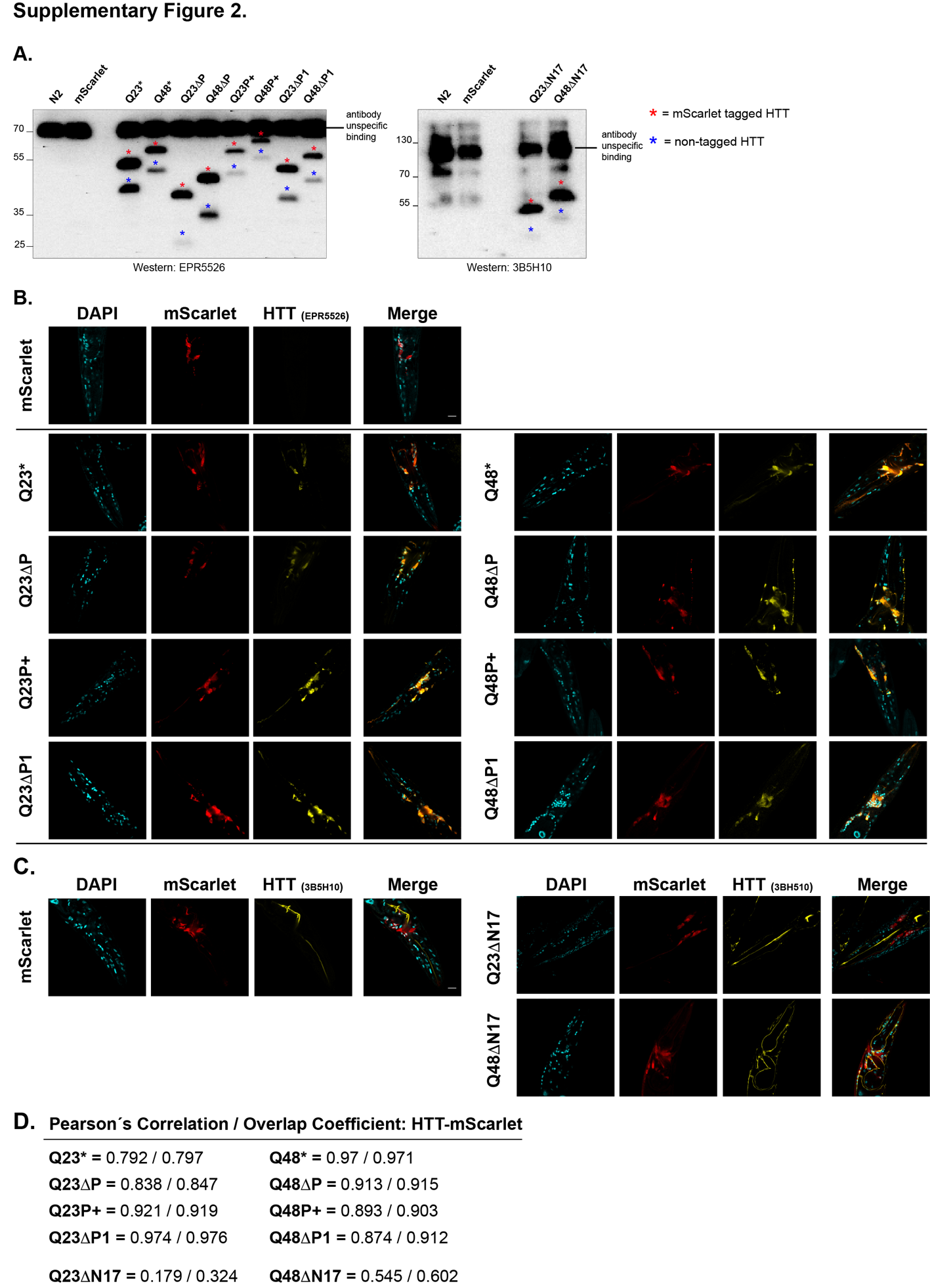
**

**A.** Western blot of HTTEx1 mutant *C. elegans* strains generated for this study confirms the double presence of HTTEx1, tagged and untagged. Left blot shows the wildtype N2 nematode, the mScarlet control and all HTTEx1 mutants except the ∆N17 variants. Right blot shows the N2 and mScarlet controls alongside the Q23∆N17 and Q48∆N17 nematode strains. Left blot was stained against HTTEx1 with the EPR5526 antibody and right blot with the 3B5H10 antibody. Untagged HTTEx1 is indicated with a blue asterisk, while mScarlet-fused HTTEx1 is indicated with a red asterisk. Unspecific bands appear around 70 kDa, and above, for both antibodies. The contrast of the blots was increased to facilitate visualization. **B-C.** Immunohistochemistry of *C. elegans* strains confirmed the presence of HTTEx1 in head neurons, for the control, ∆P, P+ and ∆P1 strains (B). For the ∆N17 mutant strains immunohistochemistry against HTTEx1 was unable to detect the protein in the neurons, and unspecific binding, as present in the control, was instead recognized (C). First row illustrates the DAPI-stained nuclei (cyan), second row illustrates the endogenous mScarlet (red), third row shows the HTT signal (yellow), and final row represents the merge of all channels. Non-tagged HTTEx1 was stained either with anti-HTTEx1 EPR5526 or 3B5H10 antibodies and does not appear in the mScarlet control. Unspecific staining is present with the 3B5H10 antibody, as demonstrated in the control mScarlet image. A test utilizing the MW8 or 5TF1-1C2 antibodies could also not detect the ∆N17 HTTEx1 constructs (data not shown). Scale bar is 10 µm. Contrast of images was increased for better visualization. **D.** Table showing the Pearson’s correlation and overlap coefficients for the mScarlet-HTTEx1 and antibody-detected HTT signals. Coefficients demonstrate a strong (r ≥ 70) or very strong (r ≥ 90) colocalization for the non-variated, ∆P, P+ and ∆P1 strains, while a weak or moderate correlation for the ∆N17 Q23 and Q48 strains respectively. Analysis was performed using the JACoP plugin in Fiji/ImageJ.

**Supplementary Fig. S3**

**
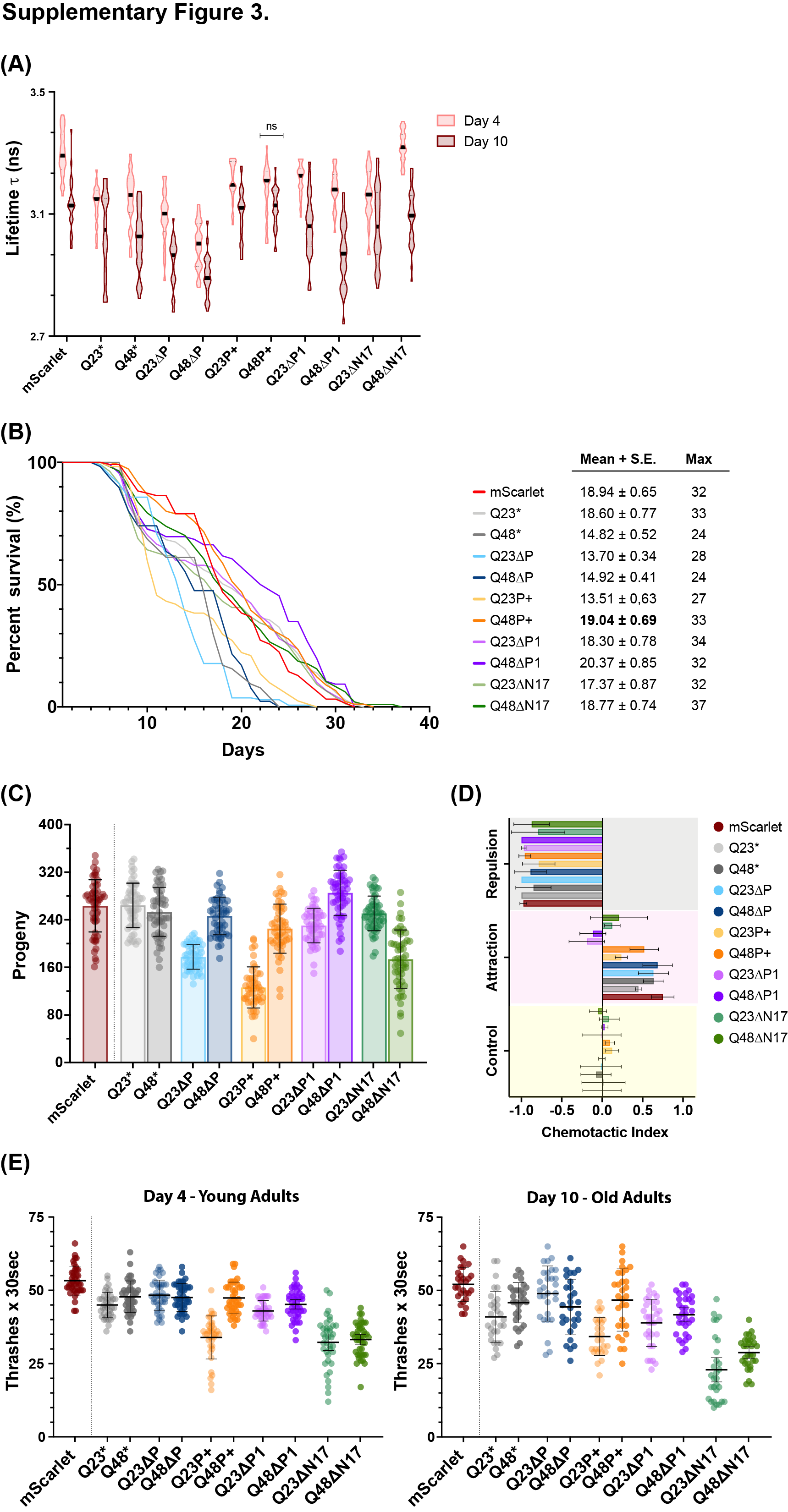
**

**A.** Truncated violin plots representing the average lifetime values of all *C. elegans* HTTEx1 strains and mScarlet control, compared between day 4 and day 10. All strains show a significant fluorescence lifetime reduction, indicating increased aggregation, between day 4 and day 10, except for Q48P+. Significance of the grouped analysis was calculated with a two-way ANOVA followed by a Sidak correction test and assuming a 95% confidence interval (ns = non-significant). **B.** Lifespan assay, illustrated with a Kaplan-Mayer plot, of all *C. elegans* HTTEx1 mutant strains alongside the mScarlet control strain. Survival curves of all *C. elegans* strains are compared here and the mean and standard error, and maximum lifespan are represented on the left. **C.** Scatter dot plot with bar of the progeny assay performed for all *C. elegans* HTTEx1 strains and mScarlet control. **D.** Grouped bar plot of chemotactic assay performed on all *C. elegans* HTTEx1 mutant strains alongside the mScarlet control strain. **E.** Scatter dot plot of the thrashing assay performed on all *C. elegans* HTTEx1 strains and mScarlet control strain, on young day 4 and on old day 10 nematodes. For all assays here depicted significance was assessed as described in figure 5 for the respective assays. Significance values are reported in Supplementary table 3 for lifespans, and Supplementary table 4 for all other phenotypic assays.

**Supplementary Figure S4.**

**
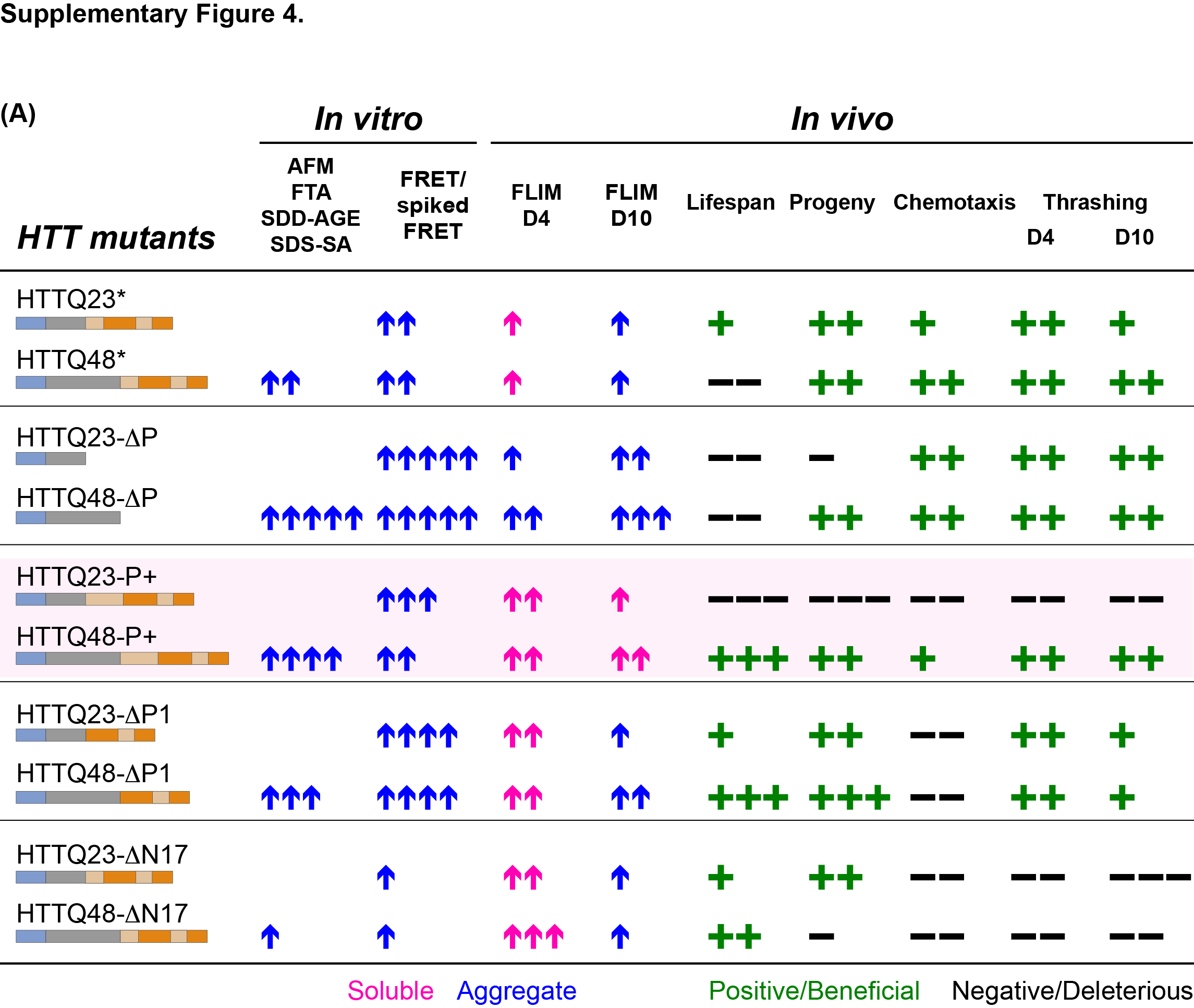
**

**A.** Summary of both the aggregation propensities for the *in vitro* HTTEx1 mutants, and of the FLIM analysis and phenotypic assays of the *in vivo* HTTEx1 *C. elegans* strains. Blue arrows represent aggregation, magenta arrows represent solubility. Number of arrows present per mutant represent the degree of aggregation/solubility. Similarly, green plus sign represents organismal advantage or positive phenotypic outcome, while black minus sign represents negative, deleterious results. Highlighted in a pink undertone are the unexpected results of the P+ mutants.
